# An exploratory fNIRS study on directional asymmetry in segmental discrimination: Lateral–rhotic (/la–ra/) and labial–dorsal (/ba–ga/) contrasts tested in 5- and 9-month-old Japanese infants

**DOI:** 10.1371/journal.pone.0358044

**Published:** 2026-09-11

**Authors:** Yoritaka Akimoto, Miki Takahasi, Naoto Yamane, Natsumi Shibata, Rachel Ka-Ying Tsui, Reiko Mazuka

**Affiliations:** 1 Information and Management Systems Engineering, Nagaoka University of Technology, Kamitomioka, Nagaoka, Niigata, Japan; 2 RIKEN Center for Brain Science, Wako, Saitama, Japan; 3 Department of Psychology and Neuroscience, Duke University, Durham, North Carolina, United States of America; Università del Salento: Universita del Salento, ITALY

## Abstract

Directional asymmetry refers to the order-dependent difference in segmental discrimination; however, its underlying cortical mechanisms remain unclear. This exploratory study used near-infrared spectroscopy to measure hemodynamic responses in 5- and 9-month-old Japanese infants (N = 30 [14 girls, 16 boys] and N = 40 [16 girls, 24 boys], respectively) to the lateral–rhotic /la–ra/ and labial–dorsal /ba–ga/ contrasts and to examine whether directional asymmetry was observed. Post hoc analysis revealed significant directional asymmetry for the /la–ra/ contrast in 5-month-old infants, characterized by decreased oxyhemoglobin levels in the right middle-anterior temporal channels, specifically for the /ra/-to-/la/ change. This localized response in the middle portion of the superior temporal gyrus, a region associated with tracking acoustic properties, suggests that infants at this age may rely on acoustic-level processing rather than on fully established phonological categories. This relative deactivation, observed in 5-month-olds but not in 9-month-olds and in the direction that appeared more difficult to discriminate, suggests a potential association between discrimination difficulty and the hemodynamic response. Specifically, the acoustic complexity of the /ra/ standard stimuli could have imposed a higher computational load, potentially leading to incomplete encoding during the baseline periods. This incomplete processing may have elevated hemodynamic activity, thereby resulting in a relative decrease in response to the subsequent change to /la/ stimuli. This phenomenon aligns with the perceptual space learning account, in which hemodynamic activity potentially reflects the computational load involved in the continuous calibration of an infant’s developing perceptual space. Future studies should directly assess this hypothesis.

## Introduction

An infant’s ability to discriminate speech sounds is crucial for language acquisition. Although infants can distinguish most phonetic contrasts, this ability changes as they age and attune to their native language. Studies have revealed that the ability to discriminate non-native consonant contrasts declines at approximately 10 months of age [1–4]. However, acoustically subtle phonetic contrasts are particularly difficult to discriminate, even for young infants [5]. Kuhl et al. [6] demonstrated that American infants exhibited a significant improvement in the discrimination of the /la–ra/ contrast between 6–8 and 10–12 months of age; however, this contrast remains highly challenging across both ages for Japanese infants, for whom it is non-native.

Additionally, Kuhl et al. [6] reported that the change from /ra/ to /la/ was more difficult to discriminate than the reverse, regardless of the infant’s native language or age group. This phenomenon, known as directional asymmetry, suggests that an infant’s ability to discriminate speech segments is affected by the direction of change. Directional asymmetry has also been observed for other contrasts [7–11]. The Natural Referent Vowel (NRV) framework [12], a prominent account of directional asymmetries, posits that infants possess a universal bias to prioritize sounds with salient articulatory or acoustic properties. This model suggests that asymmetry occurs because a change toward a more “salient” sound is easier to detect. Specifically, it emphasizes acoustic “focalization,” whereby adjacent formants closely converge to create a robust spectral peak, as a key driver of salience that attracts infant attention. Recent empirical evidence suggests that such acoustic-bias accounts may also apply to consonant discrimination. Specifically, Nam and Polka [9] discussed the possibility that this focalization principle could be applied to the /r/–/l/ contrast and proposed that the converging the second and third formants (F2 and F3) at the onset of /r/ might make it more acoustically salient than /l/. They also argued that such a salient landmark induces a phenomenon known as “perceptual clinging,” an attentional state wherein an infant’s attention becomes captivated by the acoustic prominence of the focalized stimulus. According to the Native Language Magnet theory expanded model [13], although younger infants heavily rely on these universal acoustic landmarks, accumulated exposure to their native language during the first year promotes neural reorganization, warping the perceptual space to align with native phonetic categories. Additionally, the perceptual space learning framework [14] posits that infants construct an appropriate perceptual space to represent speech sounds rather than forming discrete phonetic categories. During this constructive process, processing acoustically complex referents imposes a higher computational load associated with the continuous calibration of the perceptual space.

Brain imaging studies have also investigated infants’ brain responses during segmental discrimination [15–22]. Previous research indicates that the bilateral superior temporal gyrus plays a crucial role in early speech perception [23]. Specifically, younger infants tend to rely on acoustic-level processing for the segmental discrimination of both native and non-native contrasts, which recruits either bilateral or right-dominant networks [24]. Over development, as these auditory cues gain linguistic significance, neural commitment undergoes a functional lateralization shift toward the left-hemispheric language network [24]. Yet, the brain mechanisms underlying directional asymmetry remain unknown.

Therefore, this exploratory study aimed to investigate whether directional asymmetry occurred in hemodynamic responses during segmental discrimination, utilizing the lateral–rhotic /la–ra/ and labial–dorsal /ba–ga/ contrasts. We selected 5- and 9-month-old Japanese infants to examine the early stage of acoustic-driven, broad phonetic sensitivity at 5 months and the emerging native-language specialization at 9 months. Although sensitivity to non-native contrasts declines at approximately 10 months, we expected infants of both ages to exhibit successful discrimination performance, as the ability to differentiate these segments persists even at 10–12 months [6,25]. First, we conducted a visual habituation–dishabituation experiment with 5-month-old Japanese infants using these contrasts. Subsequently, we measured the hemodynamic responses of 5- and 9-month-old Japanese infants in the bilateral superior temporal regions using near-infrared spectroscopy (NIRS). Since previous studies have reported directional asymmetry for the /la–ra/ contrast, but not for the /ba–ga/ contrast, we anticipated observing asymmetry only for the /la–ra/ contrast and expected the /ba–ga/ contrast to serve as a control.

## Materials and methods

### Research environment and ethics statements

This study was conducted in Wako City, Saitama Prefecture, Japan. Participants were recruited from the general population in the Tokyo metropolitan area, an urban region characterized by relatively high access to education and healthcare and a generally middle-class socioeconomic profile. All parents provided written informed consent before the experiment, and all experiments were approved by the ethics committees of RIKEN (approval numbers: Wako3, 24−11(13), March 25, 2015; Wako3, 24−11(14), May 20, 2016; Wako3, 30−2(20), October 13, 2023). Parents reported that their infants had no hearing concerns and were being raised in monolingual Japanese-speaking environments. Participants were recruited from October 5, 2015, to June 27, 2017, with additional recruitment for the NIRS experiment conducted from May 31 to June 26, 2024. After the artifact-processing procedure was refined, the initial dataset met the target sample size; nevertheless, the results include valid NIRS data from five additional 9-month-old infants. Excluding these additional data did not alter the statistical outcomes for the 9-month-old NIRS dataset.

### Behavioral experiment

#### Participants.

This experiment included 102 full-term 5-month-old Japanese infants who participated in a habituation–dishabituation experiment using either the /la–ra/ or /ba–ga/ contrasts. Of these, 39 were excluded due to crying (n = 28), technical problems (n = 3), experimenter error (n = 1), or failure of habituation (n = 7; they completed 28 trials without meeting the 65% habituation criterion). Consequently, 34 infants (15 girls, mean age = 161.8 days, age range = 151–180 days) and 29 infants (15 girls, mean age = 165.5 days, age range = 152–180 days) completed the /la–ra/ and /ba–ga/ experiments, respectively. For the /la–ra/ contrast, 18 and 16 infants were assigned to the /la/ to /ra/ and /ra/ to /la/ directions, respectively. For the /ba–ga/ contrast, 14 and 15 infants were assigned to the /ba/ to /ga/ and /ga/ to /ba/ directions, respectively. Recruitment continued until each contrast included 28 infants with valid data; this target sample size was consistent with those in previous studies that used similar methods to investigate native and non-native consonant discrimination in Japanese infants (see Lovčević & Tsuji [25] for a power analysis). Considering this study’s exploratory nature, no formal power analysis was performed, and the sample size per condition (i.e., direction) followed conventional standards in infant looking-time research, where approximately 12 infants per cell were considered acceptable.

#### Stimuli.

Stimuli comprised 40 tokens, 10 each of the /la/, /ra/, /ba/, and /ga/ speech sounds, spoken in an adult-directed style by 10 native English female speakers. We used adult-directed speech to focus on segmental and directional contrasts, avoiding unintended influences from pitch and intonation, which are often exaggerated in infant-directed speech. These stimuli were selected through the following procedure. First, more than 10 tokens of each syllable were recorded from each speaker, and their intensities were normalized. After noisy or atypical tokens were excluded, the F3 transitions of the /la/ and /ra/ tokens were visually checked using Praat (Phonetic Sciences, University of Amsterdam, the Netherlands). This confirmed that the /ra/ tokens exhibited a characteristic rising F3 transition, whereas the /la/ tokens maintained a stable F3 at a higher frequency range—critical parameters for distinguishing /l/ and /r/. Subsequently, one token was selected from each syllable for each speaker to ensure that the acoustic properties, other than the critical spectral parameters, did not differ significantly among the syllable types. These properties included duration, pitch-related features (mean, maximum, and minimum pitch, and pitch range calculated as [maximum pitch – minimum pitch]), and intensity-related features (mean, maximum, and minimum dB, and intensity range calculated as [maximum dB – minimum dB]). Consequently, no significant differences were observed across the syllables, except for maximum intensity, which was slightly but significantly higher in /la/ (mean = 63.81, SE = 0.17) and /ra/ (mean = 63.77, SE = 0.14) than in /ga/ (mean = 63.20, SE = 0.20). However, this difference did not interfere with the examination of directional asymmetry, as comparisons were made only within each contrast direction and not across contrasts. Regarding the critical phonemic contrast between /la/ and /ra/, acoustic analysis confirmed that the selected /ra/ tokens exhibited a characteristic rising F3 transition (mean F3: 1944 Hz [SE = 71] at 50 ms and 2342 Hz [SE = 66] at 150 ms from onset); conversely, the /la/ tokens remained stable at a substantially higher range (mean F3: 2999 Hz [SE = 48] at 50 ms and 2958 Hz [SE = 84] at 150 ms). For the /ba–ga/ contrast, acoustic analysis confirmed a prominent focalization of formants in the /ga/ tokens at the syllable onset, characterized by a typical “velar pinch” with F2 and F3 closely converging (mean F2: 1566 Hz [SE = 52]; mean F3: 2576 Hz [SE = 69] at 50 ms). In contrast, the /ba/ tokens exhibited no such convergence, and F2 and F3 remained distinctly separated (mean F2: 1291 Hz [SE = 38]; mean F3: 2717 Hz [SE = 82] at 50 ms). Average durations of the speech tokens were 343.3 ms (SE = 20.5), 361.3 ms (SE = 18.3), 386.4 ms (SE = 21.5), and 383.7 ms (SE = 17.0) for /ba/, /ga/, /la/, and /ra/, respectively. The mean fundamental frequencies (F0s) values were 193 Hz (SE = 6), 193 Hz (SE = 5), 186 Hz (SE = 6), and 184 Hz (SE = 7), respectively.

#### Procedure.

All experiments were conducted in a sound-attenuated, dimly lit room. Infants sat on their parents’ laps in a chair positioned 1 m away from a 19-inch monitor (Hewlett-Packard L1950). A speaker (Reveal, TANNOY, Scotland, UK) located behind the monitor presented the stimuli at approximately 60 dB sound pressure level (SPL), as confirmed by a sound level meter positioned at the infant’s ear level. An experimenter monitored the infants’ visual responses from an observation room via video. Their responses were simultaneously recorded using a digital recorder for subsequent video coding. The parent listened to music through headphones to mask the auditory stimuli presented to the infants.

A modified visual-habituation paradigm [25 –26] was used to measure infants’ speech discrimination abilities. An attention-getter (a brightly colored moving chick) appeared on the screen before each trial. The experimenter pressed a key to begin the next trial once the infant looked at it. In each habituation phase trial, infants were repeatedly exposed to one stimulus (e.g., /la/). Each trial lasted 15 seconds, and 15 speech tokens were presented with a 1-second stimulus onset asynchrony. Tokens were randomly drawn from a set of 10; five appeared twice and the remaining five once, which resulted in 15 tokens being presented in a randomized order. Degree of habituation was assessed by measuring the looking time at a visual checkerboard that appeared concurrently with the presentation of an auditory stimulus. The checkerboard was a static red-and-black image that remained on the screen throughout each trial. The cumulative looking time was measured by the experimenter when the infant looked at the visual checkerboard. The test phase began when the average looking time in the last four habituation trials reached less than 65% of that in the first four habituation trials or when 28 trials were completed. Each infant completed one change trial and one no-change trial, with the order counterbalanced across participants. In the no-change trials, 15 speech tokens of the same syllable as in the habituation phase (e.g., /la/) were presented, whereas a different syllable (e.g., /ra/) was presented in the change trials. Infants’ looking times were manually coded from the video recordings on a frame-by-frame basis by trained coders blinded to the experimental conditions. The total duration of gaze directed at the checkerboard stimulus was calculated for each trial.

### NIRS experiment

#### Participants.

In total, 44 full-term 5-month-old and 58 full-term 9-month-old Japanese infants participated in two fNIRS sessions (i.e., both the /la–ra/ and /ba–ga/ sessions, in either direction). Each session lasted less than 5 minutes, typically with approximately 2-minute intervals between sessions, during which the experimenters ensured that the infant was calm and comfortable. The subsequent session commenced once the infant and system were ready. Infants who participated in the behavioral experiment did not participate in the NIRS experiment. Data from 14 5-month-olds and 18 9-month-olds were excluded due to crying, refusal to wear probes, hair obstruction, and/or large motion artifacts. Of these, 10 infants were excluded because they did not complete the sessions (eight 5-month-olds and two 9-month-olds). An additional 22 infants were excluded due to an insufficient number of valid channels within the regions of interest (ROI): six 5-month-olds (three in one session and three in both sessions) and 16 9-month-olds (nine in one session and seven in both sessions); see the NIRS Analysis section for the criteria. The final sample comprised 30 5-month-olds (14 girls; mean age = 168.2 days, range = 153–183 days) and 40 9-month-olds (16 girls; mean age = 287.9 days, range = 274–304 days). Of these, 29 infants (23 5-month-olds and six 9-month-olds) participated in this study following another NIRS study (typically lasting < 10 minutes). In the 5-month-old group, 14, 16, 16, and 14 datasets were valid for the changes from /la/ to /ra/, /ra/ to /la/, /ba/ to /ga/, and /ga/ to /ba/, respectively. In the 9-month-old group, the numbers were 19, 21, 23, and 17, respectively.

Both age groups were recruited in parallel, and recruitment continued until at least 28 infants per group had valid data. Owing to the exploratory nature of this study, no formal power analysis was conducted. Sample sizes were determined based on previous studies and practical considerations. Our final sample size was within the typical range for infant NIRS research, where group sizes typically had a median of 15 and rarely exceeded 40 participants [27]. Additionally, comparable studies that used the same NIRS equipment and similar experimental paradigms [24, 28] reported final sample sizes that ranged from 19–24 infants per age group.

#### Stimuli.

The same /la/, /ra/, /ba/, and /ga/ stimuli as those used in the behavioral experiment were used.

#### Procedure.

**Fig 1:**
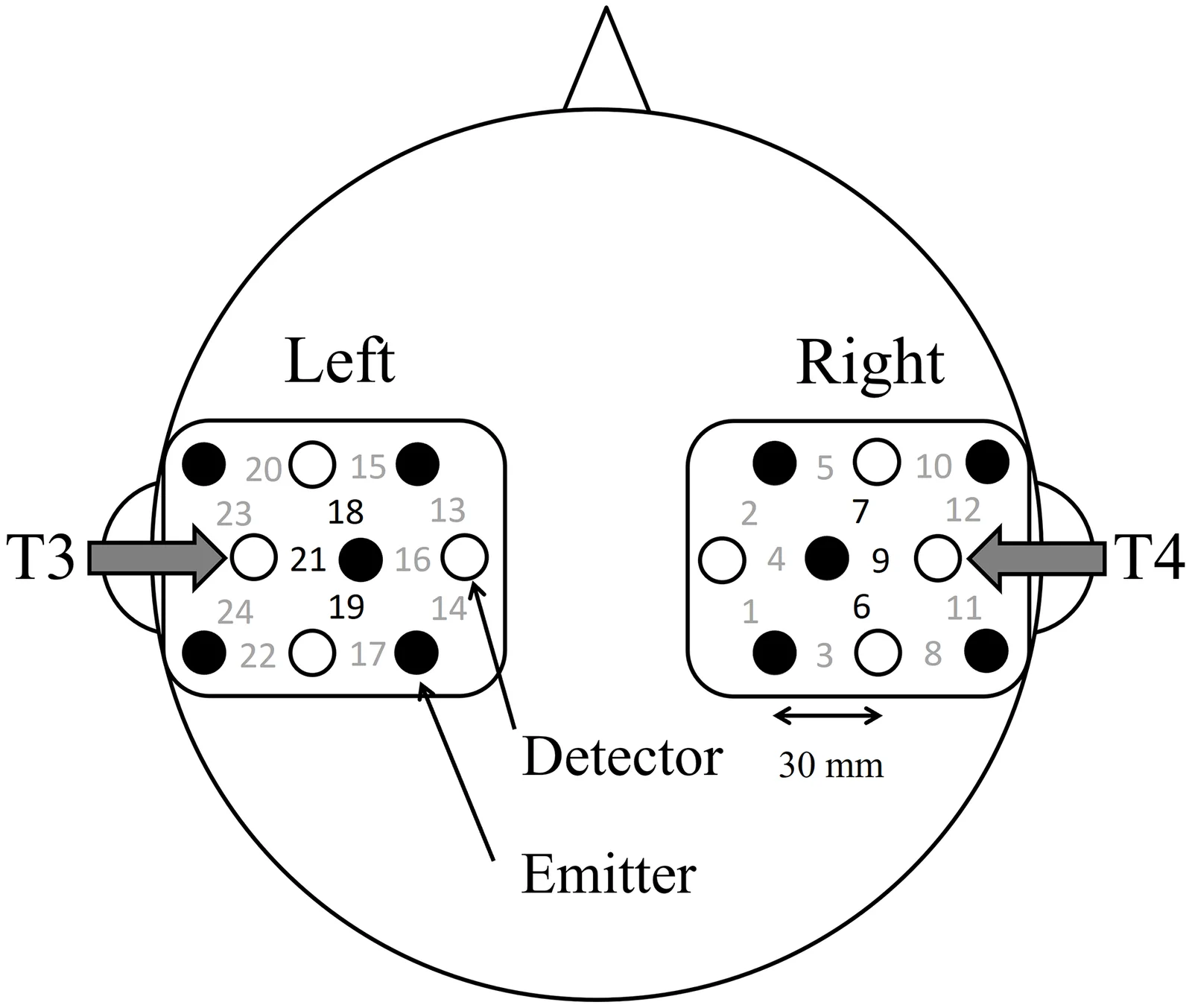
Placement of the near-infrared spectroscopy probes.

Hemodynamic responses were measured at a sampling rate of 10 Hz via a multichannel NIRS system (ETG-4000, Hitachi Medical Corp., Tokyo, Japan) that used near-infrared light with wavelengths of 695 and 830 nm. The placement of the NIRS probes was similar to that in previous studies [23,24,28]: five emission and four detection probes were attached to each lateral side of the head (Fig 1). Mid-bottom detector probes for the left and right sides were placed near the T3 and T4 positions, respectively. The distance between the emission and detection probes was 3 cm.

Black and white circles represent the emitter and detector, respectively. Measurement channels are numbered. Channels 6, 7, 9, 18, 19, and 21 are defined as regions of interest.

Additionally, eight emission probes and seven detection probes were attached to the frontal region, with the vertical midline and lowest line corresponding to the nasion–inion and Fp1–Fp2 lines, respectively. However, these were not analyzed, as they were considered not directly relevant to the directional asymmetry of consonant perception.

Infants were seated on a parent’s lap in a sound-attenuated room. Stimuli were presented at approximately 60 dB SPL from a loudspeaker (Reveal, TANNOY, Scotland, UK) located 70 cm in front of the infant, as confirmed via a sound level meter positioned at the infant’s ear level. The parent and one or two experimenters, who listened to music through headsets for masking, silently entertained the infant with toys. A silent movie was also played on the monitor.

**Fig 2:**
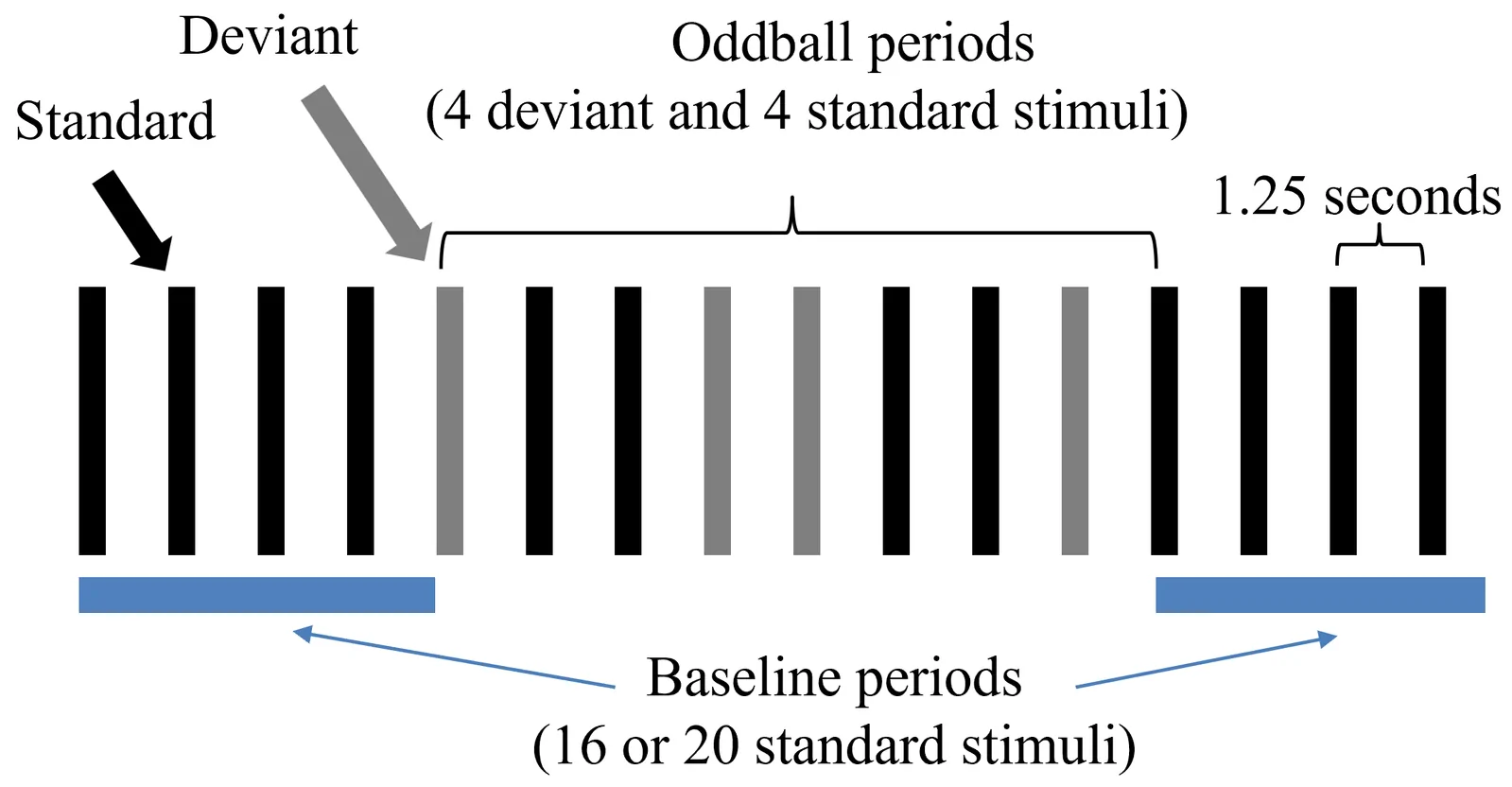
Experimental design of the fNIRS sessions using a modified oddball paradigm. Baseline and oddball periods alternated, with sounds presented every 1.25 seconds throughout. Baseline periods (20 or 25 s, randomly determined) included only standard sounds; conversely, oddball periods (10 s) included four standard and four deviant sounds in a pseudorandom order, always beginning with a deviant.

Each infant participated in two fNIRS sessions via a modified version of the oddball design [24], one each for the /la–ra/ and /ba–ga/ contrasts in either direction (Fig 2). Standard sounds were presented every 1.25 seconds during the baseline periods, which lasted either 20 or 25 seconds (randomly determined), resulting in 16 or 20 tokens per baseline period. These baseline periods alternated with oddball periods that lasted 10 seconds, during which both standard and deviant sounds were presented in a pseudorandom order with equal probability, resulting in 8 tokens per oddball period (4 standard and 4 deviant). The first stimulus in each oddball period was always deviant. The onset timing of the tokens was kept constant at 1.25 seconds throughout both periods, without any additional silent intervals. Seven alternations were presented, if possible. The order of the contrast type (i.e., /la–ra/ or /ba–ga/) and direction (i.e., assignment of consonants as standard or deviant) was counterbalanced across participants.

### Analysis

fNIRS data were analyzed via the HOMER3 package version 1.80.2 [29]. Channels 6, 7, and 9 for the right hemisphere and 18, 19, and 21 for the left hemisphere (Fig 1) were defined as ROIs, which presumably covered the superior temporal regions [23,24,28]. The devfOLD toolbox [30,31] confirmed this assumption. After noisy channels with a low signal-to-noise ratio were excluded via “hmrR_PruneChannels,” with the parameter SNRthresh = 2, raw light intensity measurements were converted to changes in optical density via “hmrR_Intensity2OD.” Motion artifacts were identified using “hmrR_MotionArtifactByChannel,” with parameters tMotion = 1.0, tMask = 1, AMPthresh = 4, and STDEVthresh = 15. Subsequently, they were corrected by spline interpolation using “hmrR_MotionCorrectSpline,” with the parameter *p* = .99. Furthermore, wavelet filtering was performed using “hmrR_MotionCorrectWavelet,” with the parameter iqr = 0.5. Residual artifacts were identified using “hmrR_MotionArtifactByChannel” with the same parameters, and trials that contained residual artifacts were excluded. These parameters were selected according to Di Lorenzo et al.’s recommendations [32]. After band-pass filtering between 0.01 and 0.09 Hz using “hmrBandpassFilt,” optical density data were converted to oxygenated (oxy-Hb) and deoxygenated hemoglobin (deoxy-Hb) concentrations using “hmrOD2Conc,” with the parameter ppf = 1. Each epoch spanned −5–25 seconds relative to the onset of the oddball periods. To remove signal drifts, baseline correction was performed by fitting a linear function to the mean signal during the initial 5 seconds (−5–0 s) and final 5 seconds (20–25 s) of each epoch. Finally, concentration data were averaged across trials from −5–25 s after onset. Infants with < 5 available ROI channels (defined as ≥ 3 available trials) were excluded.

To examine directional asymmetry in hemodynamic responses, we primarily analyzed oxy-Hb concentrations; however, deoxy-Hb was also examined for completeness. We calculated the mean concentration changes over a 20-second window from the onset of the oddball periods and averaged the data across the ROI channels for each hemisphere. Subsequently, Welch’s two-tailed unpaired t-tests were conducted to compare the directional conditions for each contrast, with Bonferroni correction applied for the four comparisons (2 contrasts × 2 hemispheres).

## Results

### Behavioral experiment

Average number of trials required for habituation was 13.3 (SE = 1.0), 16.8 (SE = 1.2), 14.9 (SE = 1.4), and 13.9 (SE = 1.2), for the directions from /la/ to /ra/, /ra/ to /la/, /ba/ to /ga/, and /ga/ to /ba/, respectively. Welch’s two-tailed unpaired t-tests revealed a significant difference in the number of trials between the /la/ to /ra/ and /ra/ to /la/ directions (t(30.26) = 2.143, *p* = .040, Cohen’s d = 0.742); more trials were required for habituation in the /ra/ to /la/ direction. In contrast, the /ba/ to /ga/ and /ga/ to /ba/ directions revealed no significant differences (t(26.14) = 0.528, *p* = .602, Cohen’s d = 0.197).

**Fig 3:**
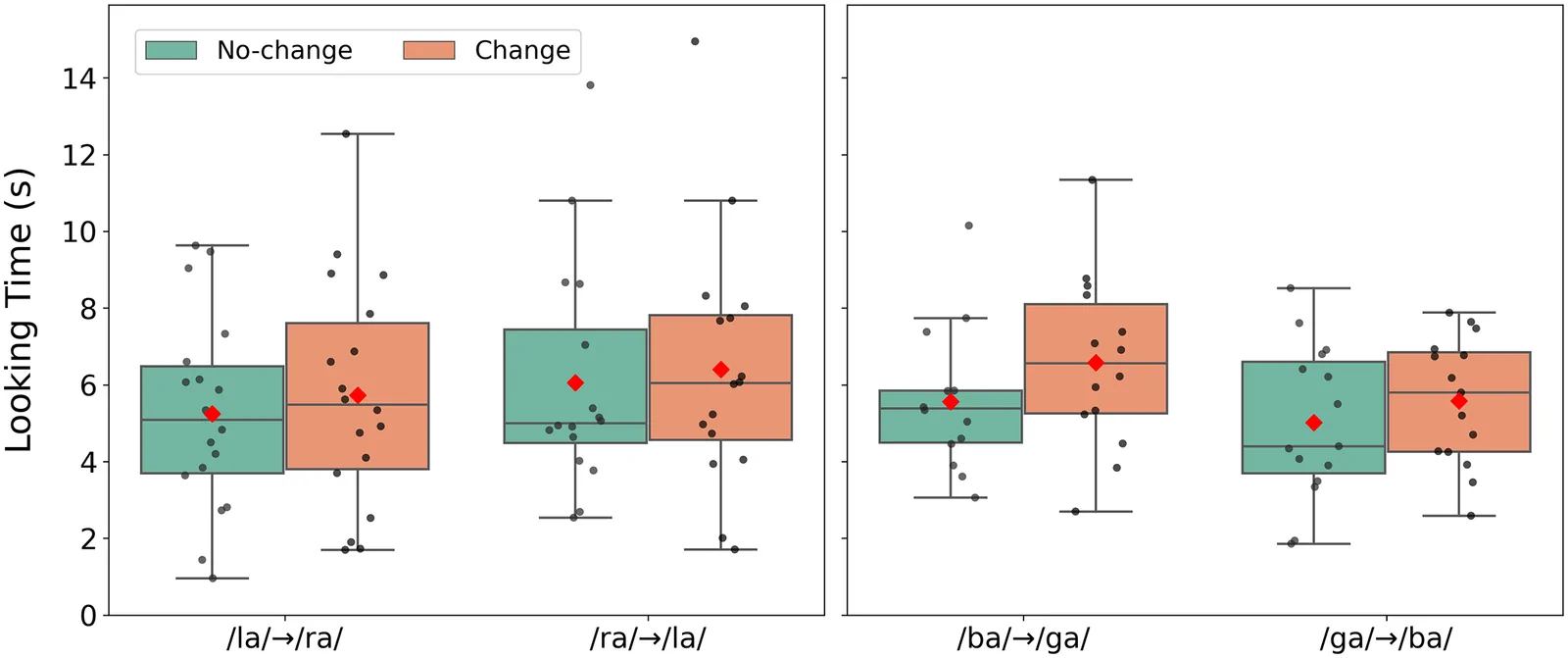
Mean looking times for each direction of the /la–ra/ and /ba–ga/ contrasts in 5-month-old infants.

Fig 3 illustrates the mean looking times for each direction of the /ba–ga/ and /la–ra/ contrasts. Considering the characteristically skewed distribution of infant looking times, all looking time data were log-transformed to ensure that the assumptions of normality for the parametric tests were met. Mixed factorial ANOVAs were conducted separately on log-transformed looking times for each contrast, with direction as the between-subjects factor and trial type (no change vs. change) as the within-subjects factor. For the /ba–ga/ contrast, the main effect of trial type was significant (F(1, 27) = 4.447, *p* = .044, $\eta_{G}^{\text{2}}$ = 0.042), which indicated that infants looked significantly longer during change trials than during no-change trials. However, neither the main effect of direction (F(1, 27) = 1.526, *p* = .227, $\eta_{G}^{\text{2}}$ = 0.040) nor the interaction between direction and trial type (F(1, 27) = 0.002, *p* = .968, $\eta_{G}^{\text{2}}$ < 0.001) was significant. No significant effects were observed for the /la–ra/ contrast. Neither the main effect of trial type (F(1, 32) = 0.345, *p* = .558, $\eta_{G}^{\text{2}}$ = 0.003) nor direction (F(1, 32) = 0.890, *p* = .353, $\eta_{G}^{\text{2}}$ = 0.021) was significant, nor was their interaction (F(1, 32) = 0.086, *p* = .772, $\eta_{G}^{\text{2}}$ < 0.001).

Red diamonds indicate condition means. Horizontal lines within the boxes represent the median. Individual data points are presented in black.

### Post-hoc exploratory analysis

Post-hoc exploratory analysis was conducted by incorporating unpublished data from a preliminary experiment that involved 9-month-old Japanese infants. These data were originally collected in a preliminary study to assess the feasibility of the experimental paradigm prior to the current study. We included these additional data to increase the overall number of observations and explore any identifiable effects within this larger, exploratory sample. The experimental procedure was the same as that for the 5-month-olds, except that 10 speech tokens were presented during each 15-second trial, with a stimulus onset asynchrony of 1.5 seconds. Tokens were presented in random order, each drawn once from a set of 10. Notably, since the stimulus intervals differed between the 5-month-old and 9-month-old experiments, this factor was not independently controlled. Consequently, any age-related effects should be interpreted as the combined effects of both age and stimulus interval differences.

The preliminary study included 51 full-term 9-month-old Japanese infants who participated in a habituation–dishabituation experiment that used either the /la–ra/ or /ba–ga/ contrasts. Of these, 22 were excluded due to failure to complete the task (crying: n = 7; other reasons: n = 2), technical problems (n = 1), experimenter error (n = 2), failure of habituation (n = 4), and extremely short looking times (< 3 seconds) in either trial during the test block (n = 6). Similarly, for this exploratory analysis, 13 additional 5-month-old infants with short looking times were excluded. The rationale for excluding infants with short looking times was that such behavior could indicate that the infant was inattentive, sleepy, or had lost interest. Recruitment continued until at least 12 infants per group had valid data. Final samples for the /la–ra/ and /ba–ga/ experiments comprised 13 (three girls, mean age = 289.3 days, age range = 274–302 days) and 16 (nine girls, mean age = 288.3 days, age range = 277–305 days) 9-month-old infants, respectively. For the /la–ra/ contrast, six and seven infants were assigned to the /la/ to /ra/ and /ra/ to /la/ directions, respectively. For the /ba–ga/ contrast, eight infants were assigned to each direction.

**Fig 4:**
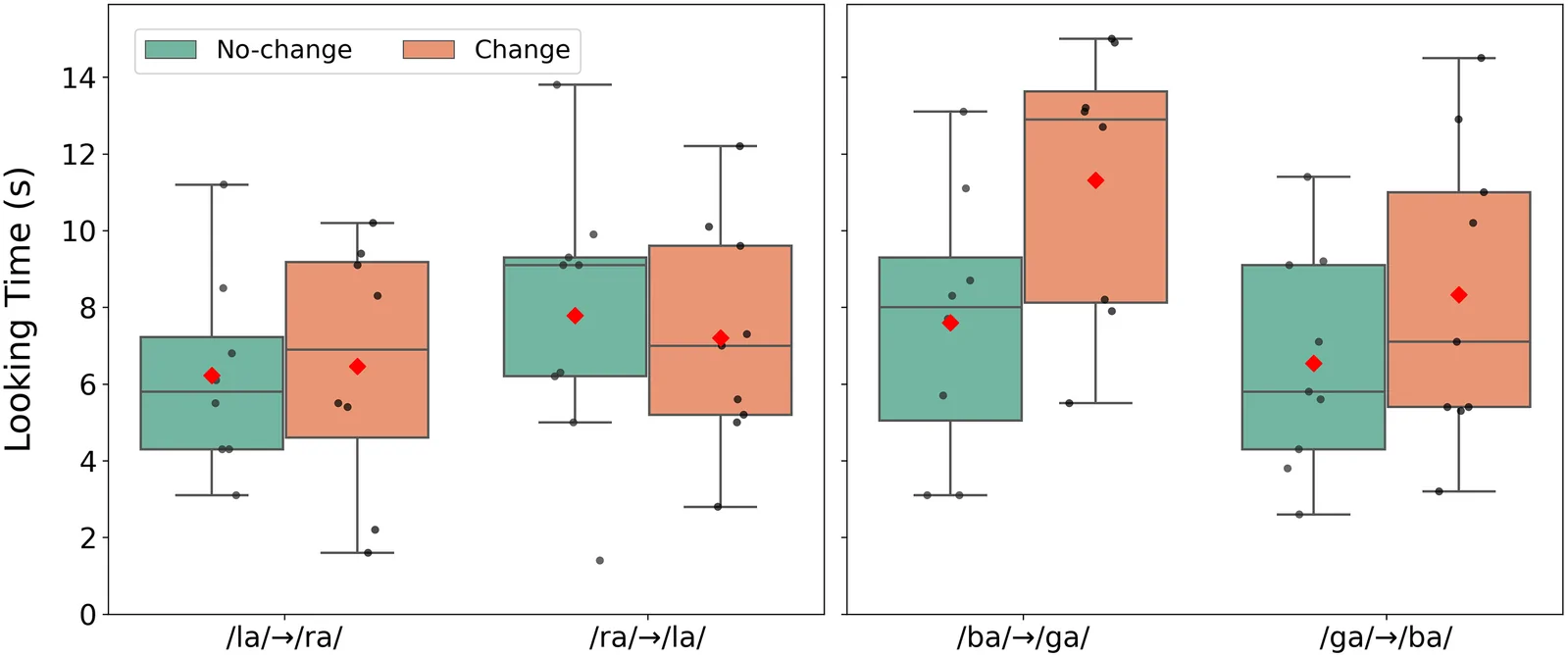
Mean looking times for each direction of the /la–ra/ and /ba–ga/ contrasts in 9-month-old infants.

Fig 4 illustrates the mean looking times for each direction of the /la–ra/ and /ba–ga/ contrasts. Mixed-factorial ANOVA was conducted on the log-transformed looking times for the /ba–ga/ contrast, with age (5- vs. 9-month-olds) and direction as between-subjects factors and trial type (no-change vs. change) as the within-subjects factor. Results revealed significant main effects of age (*F*(1, 38) = 10.294, *p* = .003, $\eta_{G}^{\text{2}}$ = 0.163) and trial type (*F*(1, 38) = 14.042, *p* < .001, $\eta_{G}^{\text{2}}$ = 0.938). Although the interaction between direction and trial type was not significant (*F*(1, 38) = 3.099, *p* = .086, $\eta_{G}^{\text{2}}$ = 0.022), we conducted simple main-effect analyses to explore the potential trends. Results revealed that the simple main effect of direction was significant only in the change trials (F(1, 38) = 4.204, *p* = .047, $\eta_{G}^{\text{2}}$ = 0.100). Specifically, the mean looking time was longer in the direction from /ba/ to /ga/ (M = 8.57, SE = 0.75) than in that from /ga/ to /ba/ (M = 6.96, SE = 0.66). Furthermore, the simple main effect of trial type was significant in the direction from /ba/ to /ga/ (F(1, 19) = 12.830, *p* = .002, $\eta_{G}^{\text{2}}$ = 0.179); looking times were longer for the change trials (M = 8.57, SE = 0.75) than for the no-change trials (M = 6.33, SE = 0.60). In contrast, this effect was not significant in the direction from /ga/ to /ba/ (F(1, 19) = 2.414, *p* = .137, $\eta_{G}^{\text{2}}$ = 0.029); mean looking times were 6.96 (SE = 0.66) and 6.09 (SE = 0.48) for the change and no-change trials, respectively.

Red diamonds indicate condition means. Horizontal lines within the boxes represent the median. Individual data points are presented in black.

Similarly, a mixed-factorial ANOVA was conducted on the log-transformed looking times for the /la–ra/ contrast, with age and direction as between-subjects factors and trial type (no-change vs. change) as the within-subjects factor. Results revealed no significant main effects of age (F(1, 33) = 1.012, *p* = .322, $\eta_{G}^{\text{2}}$ = 0.016), direction (F(1, 33) = 0.965, p = .265, $\eta_{G}^{\text{2}}$ = 0.021), or trial type (F(1, 33) = 1.208, p = .280, $\eta_{G}^{\text{2}}$ = 0.017). Although the three-way interaction was not significant (F(1, 33) = 2.956, *p* = .095, $\eta_{G}^{\text{2}}$ = 0.040), we conducted follow-up two-way ANOVAs (age × trial type) separately for each direction to explore potential trends. No significant main effects of trial type (F(1, 16) = 3.736, *p* = .071, $\eta_{G}^{\text{2}}$ = 0.098) or age (F(1, 16) = 0.008, *p* = .927, $\eta_{G}^{\text{2}}$ < 0.001) were found, nor was an interaction observed for the /la/ to /ra/ direction (F(1, 16) = 1.552, *p* = .231, $\eta_{G}^{\text{2}}$ = 0.043). Similarly, no significant main effects of trial type (F(1, 17) = 0.122, *p* = .733, $\eta_{G}^{\text{2}}$ = 0.003) or age (F(1, 17) = 2.235, *p* = .153, $\eta_{G}^{\text{2}}$ = 0.066) were found, nor was their interaction observed for the /ra/ to /la/ direction (F(1, 17) = 1.424, *p* = .249, $\eta_{G}^{\text{2}}$ = 0.037).

### NIRS experiment

Among the 5-month-old infants, the average number of analyzed trials was 6.9 (SE = 0.1), 6.4 (SE = 0.3), 6.6 (SE = 0.2), and 6.7 (SE = 0.2), for the directions from /la/ to /ra/, /ra/ to /la/, /ba/ to /ga/, and /ga/ to /ba/, respectively. Among the 9-month-old infants, these values were 6.3 (SE = 0.3), 6.4 (SE = 0.3), 6.2 (SE = 0.3), and 6.8 (SE = 0.2), respectively. Average percentages of discarded channels were 1.7% and 2.5% for 5- and 9-month-old infants, respectively.

**Fig 5:**
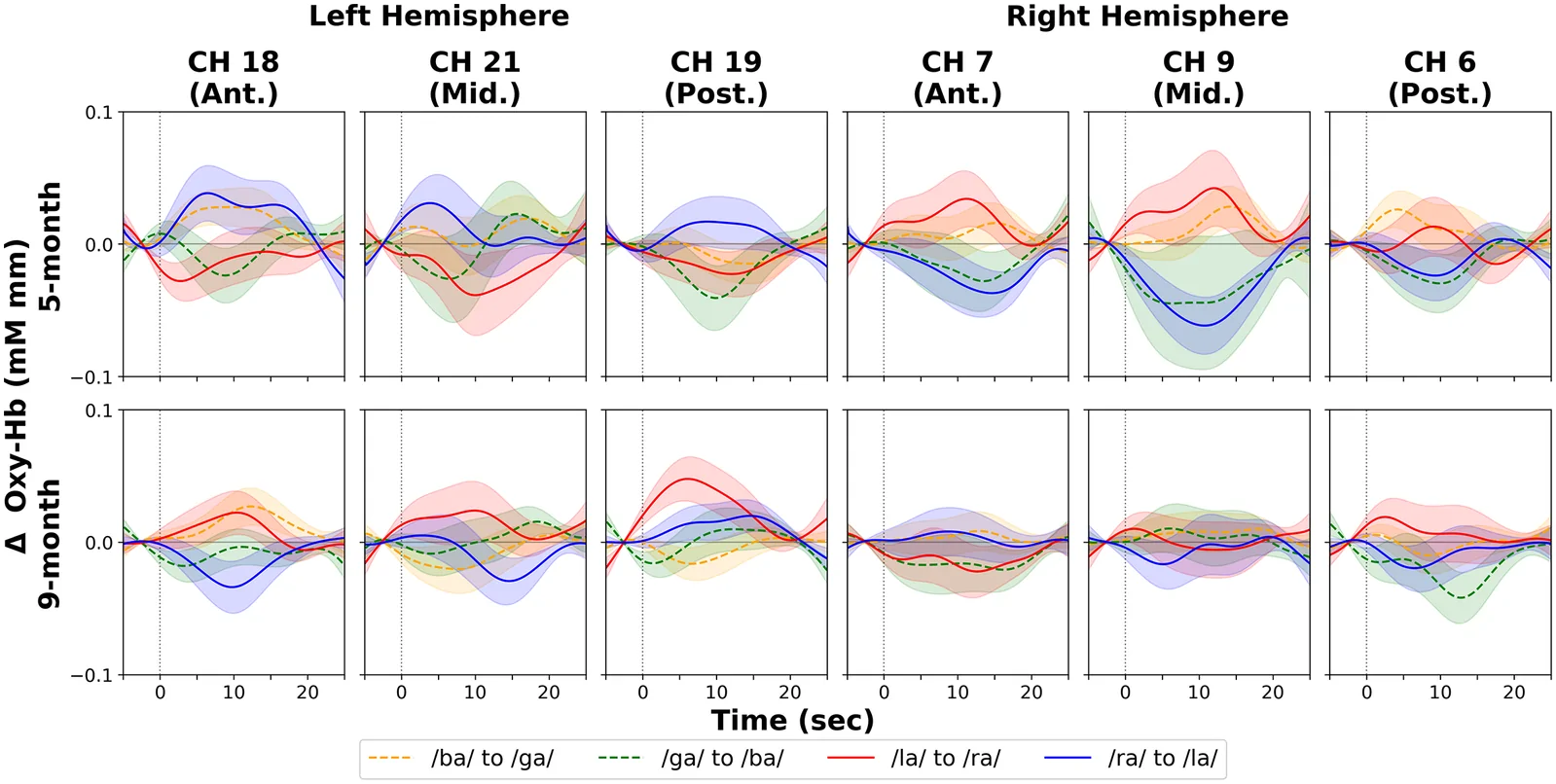
Time Course of Oxy-Hb at the individual channels within the ROIs for the /la–ra/ and /ba–ga/ contrasts in each direction.

**Fig 6:**
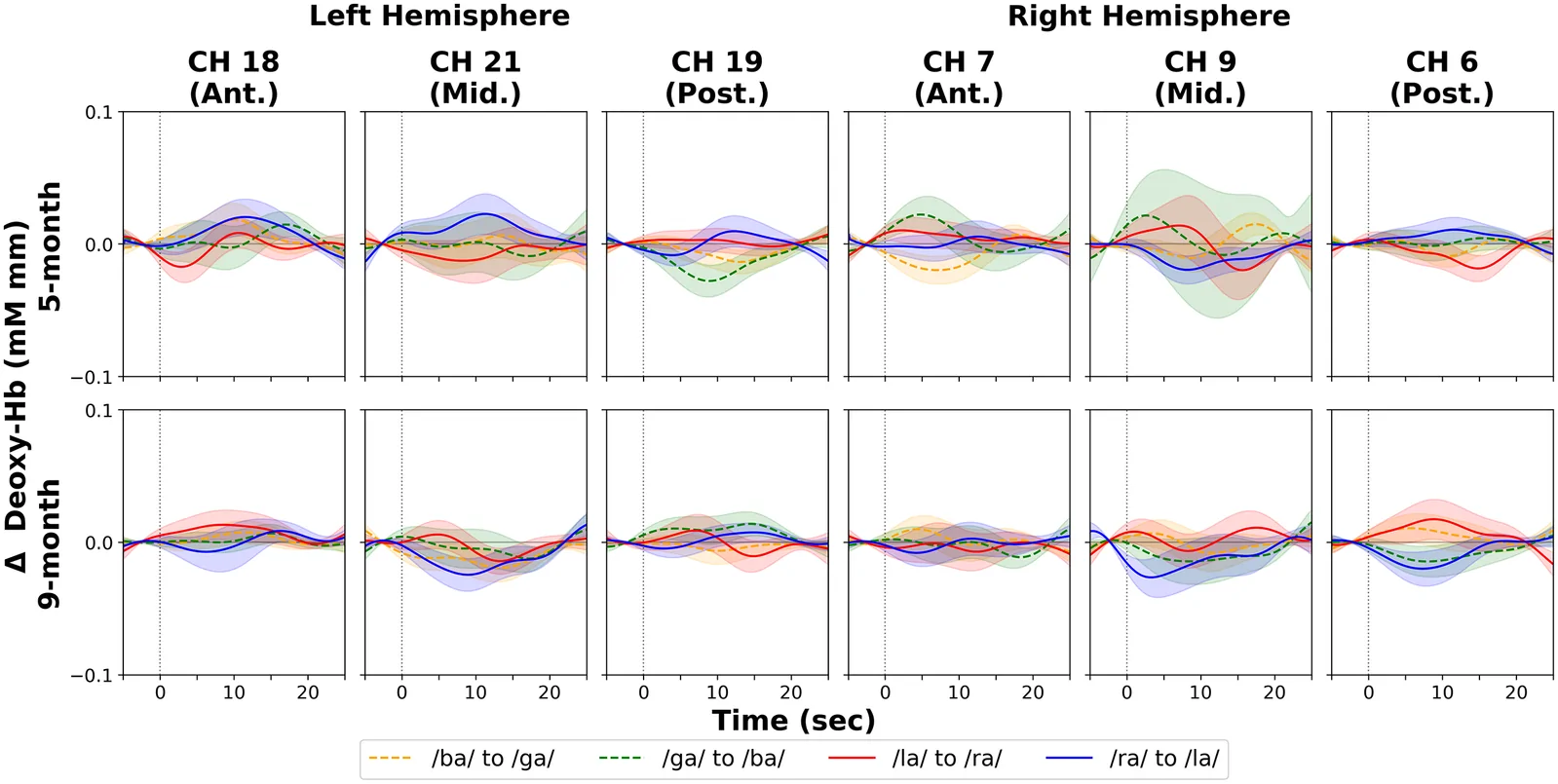
Time course of deoxy-hb at the individual channels within the ROIs for the /la–ra/ and /ba–ga/ contrasts in each direction.

**Fig 7:**
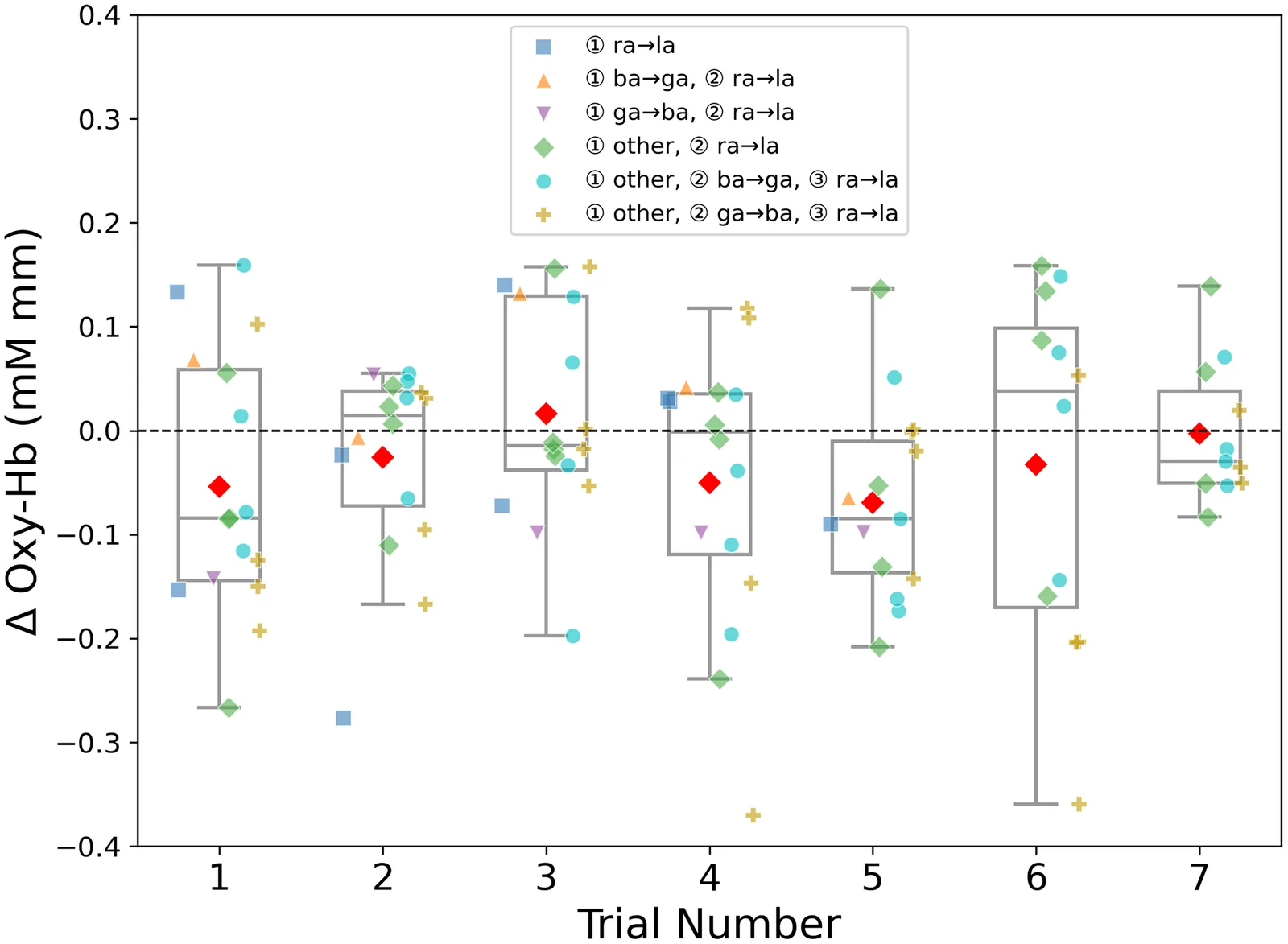
Single-trial Oxy-Hb responses for the /ra/ to /la/ change averaged across the right middle and anterior channels.

Figs 5 and 6 illustrate the time courses of oxy-Hb and deoxy-Hb responses at the individual channels within the ROIs for each directional condition in each age group, respectively. Analysis of the data averaged across the entire ROI revealed no significant directional effects for any contrast in either age group after applying Bonferroni corrections; however, visual inspection of the waveforms for 5-month-olds suggested a trend toward deactivation specifically within the middle and anterior channels of the right hemisphere, particularly for the /ra/ to /la/ and /ga/ to /ba/ directional changes. To further investigate this localized trend, we conducted a post-hoc analysis that focused on the average of the middle and anterior channels. After Bonferroni correction (2 contrasts × 2 hemispheres) was applied, analysis revealed a significant effect specifically in the right hemisphere of 5-month-olds: oxy-Hb concentrations were significantly lower for the /ra/ to /la/ change compared with the /la/ to /ra/ change (t(23.90) = 2.843, Bonferroni-corrected *p* = .036, Cohen’s d = 1.061). Fig 7 presents the single-trial oxy-Hb responses for the /ra/-to-/la/ change averaged across the right middle and anterior channels. Boxplots illustrate the trial-by-trial distribution and highlight a transient pattern in which the mean responses remained predominantly negative during the middle stages of the session (Trials 4–6) before recovering in Trial 7. Visual inspection of the individual data points, categorized based on prior experimental history, indicated that the distribution of responses showed no obvious systematic bias related to the infants’ previous experimental experience. For the /ba–ga/ contrast, the directional difference was not significant after correction (t(27.99) = 2.371, uncorrected *p* = .025, Bonferroni-corrected *p* = .099, Cohen’s d = 0.861).

Lightly shaded areas around the traces indicate the standard error.

Lightly shaded areas around the traces indicate the standard error.

Boxplots illustrate the distribution of responses across Trials 1–7. Red diamond markers indicate the mean response for each trial, and horizontal lines within each box represent the median. Individual data points are superimposed, with different colors and shapes representing each infant’s experimental history. The legend specifies the sequence and content of prior experimental history.

### Bayesian analyses

To address concerns regarding the relatively small sample sizes and to quantify the strength of these findings beyond traditional p-values, we conducted complementary Bayesian analyses using the Pingouin library (version 0.5.3) in Python. We calculated Bayes factors (BF_10_) for the primary t-tests using a Jeffreys-Zellner-Siow prior (Cauchy distribution, scale = 0.707) based on Rouder et al.’s framework [33]. Resulting BF_10_ values were interpreted according to the classification scheme proposed by Lee and Wagenmakers [34], in which values of 3–10 and 0.10–0.33 indicate moderate evidence for the alternative and null hypotheses, respectively. Conversely, values between 0.33 and 3 indicate anecdotal evidence, which is considered insensitive.

Regarding the post-hoc exploratory analysis of the behavioral results, Bayesian paired t-tests were conducted for each age group and change direction to compare the log-transformed looking times between the change and no-change trials. Among 5-month-old infants, the BF_10_ was 0.722 for the /ba/ to /ga/ change, indicating anecdotal evidence. In contrast, moderate evidence for the null hypothesis (absence of discrimination) was found for the /ga/ to /ba/, /la/ to /ra/, and /ra/ to /la/ change trials (all BF_10_ < 0.27). Among 9-month-old infants, the BF_10_ values ranged from 0.368 to 0.993, indicating anecdotal evidence.

For the NIRS data, the mean oxy-Hb concentrations in the right middle-anterior channels identified in the post-hoc analysis were analyzed. A comparison of the /la/ to /ra/ and /ra/ to /la/ directions among 5-month-old infants yielded a BF_10_ of 5.883, providing moderate evidence for the existence of directional asymmetry. For the /ba/ to /ga/ vs. /ga/ to /ba/ contrast, the BF_10_ was 2.605, indicating anecdotal evidence. In contrast, for 9-month-old infants, the BF_10_ values for the corresponding right-hemisphere comparisons were 0.310 and 0.320 for the /la–ra/ and /ba–ga/ contrasts, respectively, both indicating moderate evidence for the absence of directional asymmetry.

## Discussion

Behavioral results suggested that Japanese infants had difficulty discriminating the /la–ra/ contrast. This was further supported by complementary Bayesian analysis, which revealed moderate evidence for the null hypothesis (absence of discrimination) in both directions in 5-month-old infants. Given that previous studies have shown that older Japanese infants (6–11 months) can discriminate the /la–ra/ contrast [6,25,35], this result likely reflects the acoustic subtlety of the /la–ra/ stimuli, rather than a complete inability to distinguish it. Unlike previous studies, which typically used either synthesized stimuli [6,35] or tokens from a single speaker [25], the present study utilized 10 tokens from 10 speakers, thereby providing greater acoustic variation.

No significant directional effect was observed in looking times for the /la–ra/ contrast, which is consistent with Lovčević and Tsuji [25]. However, a significant asymmetry emerged during the habituation phase, where infants required more trials to habituate to /ra/ than to /la/. This finding aligned with that of Kuhl et al. [6], who reported that 81% of infants who failed the training criterion were in the /ra/ background condition. This suggests that such asymmetry may manifest more clearly as a difference in encoding difficulty during the habituation phase, rather than as a difference in dishabituation behavior. Since acoustic variability in /r/ production has been well documented [36], this prolonged habituation may reflect an extended encoding process required to construct a stable internal representation. This notion aligns with the perceptual space learning framework [14], which posits that phonetic categories in naturalistic speech overlap substantially in acoustic space, with the degree of overlap varying across different categories. Since /ra/ tokens exhibit a dynamic rising F3 transition, whereas /la/ tokens maintain a stable F3, this dynamic movement causes /ra/ to occupy a wider, more overlapping area within the acoustic space. Consequently, structuring the developing perceptual space to accommodate the wide distribution of /ra/ imposes a continuous and higher computational load, which may delay the completion of habituation.

This study observed no significant directional effect for the /ba–ga/ contrast in either habituation trials or looking times. However, the results revealed a more nuanced picture. The frequentist analysis revealed a significant main effect of trial type in 5-month-old infants with no significant interaction; however, the complementary Bayesian analysis found moderate evidence for the null hypothesis (absence of discrimination) specifically for the /ga/ to /ba/ change, while the data for the /ba/ to /ga/ change were inconclusive. This trend was mirrored in the post-hoc exploratory analysis, which suggested longer looking times for the /ba/ to /ga/ change than for the /ga/ to /ba/ change, indicating greater difficulty in discriminating the /ga/ to /ba/ directional change. This potential directional asymmetry is consistent with the NRV framework [12]; indeed, our acoustic analysis confirmed that /ga/ tokens possessed a prominent focalization of formants (the “velar pinch”), which served as a salient acoustic landmark. Although such labial–dorsal asymmetry has not been previously reported in infant speech discrimination, labial–coronal asymmetry is well known [11,37–39]. Furthermore, labial–dorsal asymmetry is well documented in the McGurk effect [40] and early speech production [41]. Therefore, the potential asymmetry observed here is plausible and consistent with previous findings on place-of-articulation asymmetries.

NIRS results revealed a significant directional asymmetry in the /la–ra/ contrast in 5-month-old infants. Although the initial ROI analysis revealed no significant effects, this asymmetry was observed when we focused on the middle-anterior channels and excluded the less-involved posterior channel. Notably, a clear decrease in oxy-Hb was observed in the right hemisphere during the /ra/ to /la/ change. Mature speech perception is typically characterized by left-hemisphere dominance, recruiting posterior temporal regions for phonological processing [42,43]. In contrast, early infant responses to speech, such as those in 5-month-olds, are often bilateral or right-dominant [44,45], reflecting a greater reliance on acoustic-level processing of phonetic contrasts. This right-hemispheric bias aligns with neuroimaging models that attribute higher spectral resolution to the right auditory cortex [46,47], which is also highly compatible with the NRV framework’s emphasis on detecting acoustic “focalization.” Furthermore, since the middle portion of the superior temporal regions closely tracks these specific acoustic properties of speech signals [48,49], this localization suggests that this deactivation reflects a cortical response to acoustic features before the establishment of left-lateralized phonological categories.

NIRS data revealed that 9-month-old infants did not exhibit any significant directional asymmetry for either contrast. Furthermore, Bayesian analysis provided moderate evidence for its absence. This suggests that the lack of significance in the older group reflects the nature of speech processing at this age, rather than a simple lack of statistical power. This shift may be driven by accumulated exposure to the native language may promote neural reorganization at 9 months of age [13]. This aligns with the findings of Tsuji et al. [39], which demonstrate that coronal–labial asymmetry is best explained by an interaction between language-general perceptual biases and language-specific experience.

Although deactivation has often been observed in infant NIRS studies, its underlying mechanism remains unclear [50]. Recent fNIRS studies on speech processing in sleeping infants [51,52] revealed two independent components: positive canonical and negative late-latency components that presumably reflect auditory processing and brain arousal responses, respectively. Lee et al. [52] also reported that the /ba/ to /ga/ change elicited the smallest amplitude of the negative component compared to less subtle contrasts. Although we also observed no deactivation in response to the /ba/ to /ga/ change, our results differed, as deactivation was observed for the /ra/ to /la/ change (and potentially the /ga/ to /ba/ change), which appeared more difficult to discriminate and was considered more subtle. Considering these discrepancies and methodological differences between their sleeping participants and our awake infants, whether the deactivation observed in this study corresponds to Lee et al.’s arousal response remains unclear.

Since significant deactivation was observed only in 5-month-old infants for the /ra/ to /la/ change, which appeared to be the most difficult, it might be related to discrimination difficulty. Although different acoustic factors may contribute to the processing of each contrast (see Nam et al. [53] for discussion on variability), a parallel, albeit statistically inconclusive, deactivation trend was observed, specifically for the /ga/ to /ba/ change. This subtle cortical pattern mirrors the behavioral results, where directional effects emerged only in the post-hoc analysis including the 9-month-old data, as well as the anecdotal evidence found exclusively for the /ba/ to /ga/ change in 5-month-olds, in contrast to the /ga/ to /ba/ change that supported the null hypothesis. Thus, although caution is warranted given this weaker statistical status, the presence of corresponding tendencies across behavioral and hemodynamic measures might suggest that the cortical mechanisms underlying these responses may be partially shared and may reflect a common processing difficulty associated with specific change directions.

If the observed deactivation indeed reflects a cortical response to such discrimination difficulty, one possible underlying mechanism to consider is repetition suppression, a phenomenon in which the brain exhibits reduced responses to repeated stimuli [54] that has also been observed in infants during auditory habituation [55]. Under this hypothesis, if the contrast was difficult to discriminate and perceived as repeated identical stimuli, this may have resulted in a decreased hemodynamic response. However, if this were the case, deactivation would be expected throughout the entire experiment rather than only during the oddball periods. This prediction directly contradicts our observed results, rendering the repetition-suppression account unlikely.

Repetition enhancement, a phenomenon in which the brain exhibits increased responses to repeated stimuli under certain conditions [54], is a more plausible explanation. In the infant looking-time paradigm, either an increase or a decrease in looking time indicates the ability to discriminate between stimuli [56]. Specifically, an increase in looking time at the novel stimuli reflects a novelty preference, whereas a decrease reflects a familiarity preference. Whether infants exhibit a novelty or familiarity preference depends on several factors, such as age, prior exposure, and task complexity [57]. Novelty preference suggests stable encoding of the previously presented stimuli, and no further learning is required; conversely, familiarity preference implies incomplete encoding that requires continued learning. Crucially, repetition enhancement typically occurs during this familiarity-preference phase when habituation is incomplete [54]. Since NIRS measures are relative to changes in concentration, repetition enhancement during the baseline period, caused by incomplete encoding of the standard stimuli, could increase the signal during baseline; this could lead to a relative decrease in the signal during the subsequent oddball period, manifesting as deactivation. As shown in Fig 7, the mean responses remained predominantly negative during the middle stages (Trials 4–6) before recovering in Trial 7. This transient pattern tentatively aligns with the proposed account, potentially suggesting that the phenomenon is most pronounced during a specific phase when the representation of the standard stimuli is still being established. Consistent with the behavioral results, this incomplete encoding process can be explained by the perceptual space learning framework [14], in which the wide acoustic distribution of /ra/ increases processing costs and delays perceptual stabilization. While this difficulty resulted in longer habituation times during the behavioral task, it likely led to incomplete encoding during the temporally fixed NIRS baseline period. The lack of a familiarity preference in the behavioral experiment could be due to procedural differences, as habituation was explicitly confirmed before the test phase in this task.

This study’s primary limitation is its exploratory nature and modest sample size compared to the recently recommended 20–32 infants per cell [58]; thus, these findings should be interpreted with caution. Furthermore, since hemodynamic asymmetry was identified through a post-hoc analysis and our interpretation of the observed deactivation remains speculative, future studies should directly assess the hypothesis that this transient deactivation reflects factors such as incomplete encoding of standard stimuli and a stabilizing perceptual space. Additionally, examining whether these asymmetries are influenced by linguistic context or other factors, such as whether the contrasts appear in isolated syllables or full-word forms, would be valuable.

Nonetheless, this study demonstrated that 5-month-old infants exhibited directional asymmetry in hemodynamic responses to the /la–ra/ contrast, characterized by a decrease in oxy-Hb in the right auditory regions for the direction that appeared more difficult to discriminate. These findings offer a new perspective on the developmental dynamics of early speech perception before the establishment of phonological categories.

### Declaration of generative AI and AI-assisted technologies in the writing process

The authors used OpenAI’s ChatGPT (free version) and Google’s Gemini (via institutional Google Workspace for Education account) to refine Python scripts and improve the readability of the manuscript’s English expressions during manuscript preparation. Subsequently, the manuscript was further revised through English proofreading services. The authors thoroughly reviewed and edited the content and take full responsibility for the published article.
