# Supplementary material for: An exploratory fNIRS study on directional asymmetry in segmental discrimination: Lateral–rhotic (/la–ra/) and labial–dorsal (/ba–ga/) contrasts tested in 5- and 9-month-old Japanese infants
Source: PLoS One. 2026 Sep 11;21(9):e0358044. doi: 10.1371/journal.pone.0358044 (PMC13567765; doi:10.1371/journal.pone.0358044)
Supplement: Supplementary file 1 [file pone.0358044.s002.docx]

Dear Reviewers,

We would like to express our sincere gratitude to the reviewers and the editor for their constructive comments and valuable suggestions on our manuscript. Their insights have been greatly helpful in significantly improving the quality and clarity of our paper.

In accordance with the reviewers’ recommendations and our re-examination of the data, we have conducted a comprehensive revision of the manuscript. The major modifications and improvements are summarized below:

Correction of Statistical Analysis: During the revision process, we re-examined our statistical approaches for both behavioral and NIRS data. For the behavioral task, considering the characteristically skewed distribution of infant looking times, we re-analyzed the data after applying a log transformation to ensure that the assumptions for parametric tests were met; note that this re-analysis did not qualitatively alter the main findings. We also identified a technical error in the t-test calculations for the NIRS ROI analysis. Consequently, while the initial ROI analysis across the three channels within the ROI no longer reached statistical significance after correcting for multiple comparisons, a post-hoc analysis focused on the middle and anterior two channels revealed a significant directional asymmetry for the /la–ra/ contrast.

Addition of Bayesian Analysis: As suggested by the reviewer, we have newly implemented a Bayesian analysis. Specifically, for the NIRS data from 5-month-old infants, a comparison of the /la/ to /ra/ and /ra/ to /la/ directions in the right middle-anterior channels provided moderate evidence for the existence of directional asymmetry. In contrast, for the 9-month-old infants, the analysis provided moderate evidence supporting the null hypothesis, indicating the absence of this asymmetry.

Stronger Theoretical Grounding: To address the core conceptual comments, we have substantially deepened the theoretical framework of the manuscript. Specifically, we have grounded our hypotheses and interpretations within the context of the Natural Referent Vowel framework and the perceptual space learning account. Furthermore, we have integrated a discussion on hemispheric asymmetries and lateralization shifts over development.

Clarification of Methodological Details: To ensure full transparency and reproducibility, we have clarified several methodological details, such as detailed information regarding the auditory stimuli. Furthermore, the related files on the Open Science Framework (OSF) have been updated and re-uploaded.

Re-English Editing: Given that these comprehensive revisions affect the entire manuscript, the entire text has been subjected to another round of professional English editing.

We believe that these extensive revisions have successfully addressed all the concerns raised by the reviewers and have significantly enhanced the theoretical and empirical value of our study. Given the comprehensive nature of these updates across the manuscript, we have not listed specific page and line numbers for each item; instead, all changes can be easily tracked in the revised manuscript with track changes, and we outline the core updates concisely in our point-by-point responses below.

*Reviewer #1: The study investigated infants' speech perception of a /r/ -/l/ vs /b/-/g/ contrast and explored the question of asymmetrical speech processing. Hereby, the authors tested two groups of infants, 5-month-olds and 9-month-olds, in a behavioural and fNIRS experimental setting. While behaviourally the results showed no asymmetrical perception of the /r/-/l/ contrast, the authors reported asymmetries in the 5 month-olds for the /l/- /r/ (with lower oxy-Hb in the direction from /r/ to /l/ in the right hemisphere) and /b/-/g/ (with lower oxy-Hb in the direction from /g/ to /b/ in the right hemisphere). No asymmetries were found in the group of 9-month-old infants*

*The study presents valuable insights into Japanese infants' perception of non-native speech sounds, contributing to our understanding of speech perception.*

*Introduction:*

*1. The introduction provides a foundation but could benefit from a more detailed discussion of the theoretical aspects for/ against asymmetrical perception.*

Response:

We appreciate this comment, which allowed us to clarify the theoretical focus of our revision. Rather than focusing on arguments for or against the mere existence of asymmetrical perception—which is generally recognized in the field—we have shifted the focus to why and how such asymmetries occur. Specifically, we have added a detailed discussion incorporating the Natural Referent Vowel framework to provide a stronger theoretical foundation for the emergence of these perceptual asymmetries.

*2. Also I am missing a deeper discussion on the brain imaging studies on asymmetries and the relation to native/nonnative perception and the lateralisation in the brain*

Response:

We have updated the Introduction to incorporate relevant neuroimaging findings on early speech perception and lateralization. Specifically, we have clarified that younger infants primarily recruit bilateral or right-dominant networks for acoustic-level processing of both native and non-native contrasts, and we have noted the general developmental shift toward left-hemispheric specialization as auditory cues gain linguistic significance. To keep the focus on our main topic, these neuroimaging perspectives have been integrated briefly within the context of our study.

*3. Please add a justification for the two ages tested in the current study*

Response:

We selected 5- and 9-month-old Japanese infants to examine the early stage of acoustic-driven, broad phonetic sensitivity at 5 months and the emerging native language specialization at 9 months. This justification has been newly added to the Introduction of the revised manuscript.

*The distinction between the explorative analysis and the other behavioural and nirs experiment is not clear to me. What was the difference and why were the two groups separated? What is the difference? First, I thought it was the age, because in the behavioural experiment, only the 5-month-olds were reported, but then also the 5-month-olds were mentioned as part of the exploratory analysis.*

Response:

We apologize for the confusion regarding the dataset structure. To clarify, the primary behavioral and NIRS experiments consist of the data intended for the current study (which includes both 5- and 9-month-olds for NIRS, and 5-month-olds for the behavioral task). In contrast, the post-hoc exploratory analysis was conducted by incorporating unpublished data from a preliminary experiment that involved 9-month-old Japanese infants. These data were originally collected in a preliminary study to assess the feasibility of the experimental paradigm prior to the current study. We included these additional data to increase the overall observations and explore any identifiable effects within this larger, exploratory sample. We have revised the manuscript to ensure that the distinction between the primary datasets and this exploratory sample is clearly defined.

*Statistical analyses: Were the looking times normally distributed? Please consider whether log transforms might be necessary from a statistical point of view, and whether the assumption of running ANOVAs (similarly, for the NIRS analysis and the used t-tests) holds true.*

Response:

We appreciate this important methodological comment. In accordance with your suggestion, and considering the characteristically skewed distribution of infant looking times, we have re-analyzed the behavioral data after applying a log transformation to ensure that the assumptions of normality for the parametric tests were met. For the NIRS analysis, the data distribution was not severely skewed like the looking times. Furthermore, we employed Welch's t-tests, which are robust against violations of statistical assumptions. Therefore, the validity of the NIRS analysis is justified without the need for additional data transformation.

*Behavioural results: why would it be the case that the interstimulus interval (and the difference between the 5mo and 9mo) is relevant for the direction of the /b/-/g/ contrast but not for the /l/-/r/ contrast?*

Response:

We apologize for the lack of clarity in our initial phrasing, which led to this misunderstanding. The difference in stimulus intervals between the 5-month-old and 9-month-old experiments applies to both the /b/–/g/ and /l/–/r/ contrasts, rather than specifically affecting one over the other. To prevent any further confusion, we have relocated and revised this description in the manuscript to clarify that this is a general methodological characteristic.

*Fnirs analysis: why was only the right hemisphere analysed?*

Response:

We regret that our previous description caused this misunderstanding. To make it explicit that we analyzed both the left and right hemispheres, we have revised the description in the manuscript as follows: "Welch’s two-tailed unpaired t-tests were conducted to compare the directional conditions for each contrast, with Bonferroni correction applied for the four comparisons (2 contrasts × 2 hemispheres)."

*Discussion: The authors mention that the stimuli and the greater acoustic variation might be one reason why 5-month-olds cannot discriminate the /r/-/l/ contrast. Did the authors conduct some acoustic analyses for comparison?*

Response:

We did not conduct a direct acoustic comparison with the stimuli from previous studies, as those exact tokens were unavailable. However, while previous studies typically used either synthesized stimuli or tokens from a single speaker, the present study utilized 10 tokens from 10 different speakers, making the greater acoustic variation logically apparent. To provide a clearer physical basis for our specific stimuli, we have added their acoustic profiles to the revised manuscript as follows: " Regarding the critical phonemic contrast between /la/ and /ra/, acoustic analysis confirmed that the selected /ra/ tokens exhibited a characteristic rising F3 transition (mean F3: 1944 Hz [SE = 71] at 50 ms and 2342 Hz [SE = 66] at 150 ms from onset); conversely, the /la/ tokens remained stable at a substantially higher range (mean F3: 2999 Hz [SE = 48] at 50 ms and 2958 Hz [SE = 84] at 150 ms)."

*The discussion ties together key findings, but could be strengthened by a deeper theoretical foundation. Emphasising why these findings are important beyond simply filling a gap in the literature would enhance the study’s broader relevance.*

Response:

We are deeply grateful for this comment, which directed us toward a much broader integration of our findings. Within the Discussion, we have significantly strengthened the theoretical foundation of our study. Specifically, we have grounded our interpretations within the context of the Natural Referent Vowel framework and the perceptual space learning account. Framing our empirical data within these theoretical perspectives has allowed us to elevate our findings beyond simply filling a literature gap, thereby clearly highlighting how our study contributes to understanding the underlying mechanisms of early speech processing.

*Reviewer #2: This exploratory study investigates the behavioral responses and neural basis of directional asymmetry in speech sound discrimination by measuring NIRS responses in 5- and 9-month-old infants to /la–ra/ and /ba–ga/ contrasts. The hypotheses are clearly grounded in prior literature and well motivated. Although the results are only partially consistent with previous findings, they are presented in a transparent and accessible manner. I also appreciate the authors’ appropriately cautious interpretation of the findings, particularly in light of the relatively small sample sizes and the likely limited statistical power.*

*Overall, I see some value in this work. The manuscript is well structured, identifies a specific gap (the neural basis of directional asymmetry) and addresses it using an appropriate method alongside a developmental comparison. Studies of this kind are particularly valuable, and empirical contributions in this area are very much welcomed, despite inherent difficulties.*

*Having said that, I have several concerns, especially regarding methodological specifications that could be addressed to strengthen the manuscript.*

*1. A major concern relates to the limited number of participants per condition, which raises questions about the robustness and interpretability of the findings. The authors themselves acknowledge this limitation in the conclusion (“Given the exploratory nature of this study, further investigations with larger sample sizes are needed…”), which is appropriate. However, this also makes it somewhat unclear what can be reliably inferred from the present data. In its current form, the study provides suggestive rather than conclusive evidence regarding the directional asymmetry. One of the more interesting aspects of the results is the apparent developmental difference, with effects observed in 5-month-olds but not in 9-month-olds. However, it is difficult to determine whether this reflects a genuine developmental change or is instead driven by limited power and/or methodological differences across age groups. The ambiguity of the behavioral results and the sample sizes (as few as 6 or 7 infants in some cases) further complicates the interpretation of the findings.*

*Given the practical constraints of infant research, it may not be feasible to substantially increase the sample size, which would be ideal. Alternatively, the authors should consider complementing their analyses with Bayesian approaches, which may help quantify the strength of evidence for (or against) the presence of effects, also regarding the developmental comparison. This could provide a more informative assessment than relying solely on null hypothesis significance testing under conditions of limited power.*

Response:

We are grateful for this valuable suggestion. We have conducted Bayesian analyses, which clarified the nuanced strength of our findings: for the behavioral data, the /ga/ to /ba/ change supported the null hypothesis, while the /ba/ to /ga/ change was inconclusive. For the NIRS data, the analysis provided moderate evidence for directional asymmetry in 5-month-olds (BF_10_ = 5.883), but moderate evidence for its absence in 9-month-olds (BF_10_ < 0.32). These details have been integrated into the revised manuscript.

*2. For reproducibility purposes and to facilitate future comparisons, it would be valuable to clearly specify some methodological aspects:*

*a. Beyond standard hearing screening, did the authors adopted any inclusion criteria regarding language exposure (e.g., monolingual Japanese environment)?*

Response:

Yes, all infant participants were raised in a monolingual Japanese-speaking environment. We have added this specification to the Participants section of the revised manuscript.

*b. On p. 8, when reporting on the stimuli the authors state: “After the noisy or atypical tokens were excluded, the third formant frequency transitions of /la/ and /ra/ tokens were visually checked via Praat (Phonetic Sciences, University of Amsterdam, the Netherlands), as it was the critical parameter to distinguish /l/ and /r/.” Could you please specify what was exactly seen for the transitions?*

Response:

To provide a clearer physical basis for our stimuli, and in response to a related comment from another reviewer, we have added these detailed acoustic profiles directly into the manuscript. Specifically, the acoustic analysis confirmed that the /ra/ tokens exhibited a characteristic rising F3 transition (Mean F3: 1944 Hz at 50 ms to 2342 Hz at 150 ms), whereas the /la/ tokens remained stable at a substantially higher range (Mean F3: 2999 Hz at 50 ms to 2958 Hz at 150 ms).

*c. p. 10 “The test phase began when the average looking time in the last four habituation trials reached <65% of that in the first four habituation trials or when 28 trials were completed.” Please specify the number of participants that met one and the other criterion.*

Response:

To clarify the participant numbers for each criterion, we have added this specification to the Participants section of the revised manuscript. Specifically, we have noted that 7 infants were excluded due to a failure of habituation (i.e., they completed all 28 trials without meeting the 65% habituation criterion), while all other included infants successfully met the 65% habituation criterion to proceed to the test phase.

*d. p. 12: “Approximately half of the infants participated in this experiment following another NIRS study.” Please clarify whether these infants were evenly distributed across the two age groups or belonged specifically to either the 5-month-old or the 9-month-old group.*

*e. P. 13. The authors state that “infants exhibited similar response patterns regardless of whether they had participated in the other NIRS experiment” and therefore the data were analyzed together. It would be important to clarify how this equivalence was assessed. Was this conclusion based on visual inspection of the data, or was it formally tested statistically? If statistical analyses were conducted, these should be explicitly reported.*

*f. P. 13 “…each infant participated in two fNIRS sessions”. Please clarify the length of each session and the time interval between these sessions. Additionally, were any analyses conducted to test for session order or carryover effects (e.g., comparing first vs. second session responses)?*

Response:

To clarify the distribution (comment d), we have specified the exact numbers in the Participants section of the revised manuscript: of the 29 infants with prior study experience, 23 were 5-month-olds and 6 were 9-month-olds. Regarding how the equivalence was assessed and whether session order or carryover effects were present (comments e and f), these conclusions were based on visual inspection of the data rather than formal statistical testing. As now illustrated in Fig 7, we categorized individual data points based on their full experimental history (including both prior study experience and session order within the current experiment). The trial-by-trial distribution indicated that the patterns of hemodynamic responses showed no obvious systematic bias related to the infants' previous experience or session order. Additionally, we have added the session lengths and intervals to the Method section of the revised manuscript as follows: " Each session lasted less than 5 minutes, typically with approximately 2-minute intervals between sessions, during which the experimenters ensured that the infant was calm and comfortable. The subsequent session commenced once the infant and system were ready."

g. P. 16 “ Infants with <5 available ROI channels (defined as ≥3 available trials) in any session were excluded. Please specify how many participants were excluded for this reason.

Response:

To specify the exact number of participants excluded based on this criterion, we have updated the Participants section of the revised manuscript with the detailed breakdown as follows: " An additional 22 infants were excluded due to an insufficient number of valid channels within the regions of interest (ROI): six 5-month-olds (three in one session and three in both sessions) and 16 9-month-olds (nine in one session and seven in both sessions); see the NIRS Analysis section for the criteria."

*h. Plot fNIRS and Plot looking time are not available in the OSF page.*

We apologize for the omission. We have now thoroughly updated our OSF repository to ensure transparency and completeness. Specifically, we have uploaded the generated plot files for both the fNIRS and looking time data, along with the corresponding plotting scripts. Furthermore, we have replaced the repository data—which previously consisted of trial-averaged values—with the single-trial data to allow for comprehensive verification and replication of our single-trial findings (such as those presented in Fig 7).

*Lastly, regarding the deactivation found: in order to confirm/disconfirm their interpretation the authors could directly explore whether the responses progressively increase throughout the study in the baseline and/or decrease in oddball periods, by checking the responses in the single trials over time (without averaging them).*

Response:

We are highly grateful for this insightful and constructive suggestion. Following your recommendation, we plotted the single-trial data over time to examine the trial-by-trial pattern. The results have been visualized in the newly added Fig 7. As shown in Fig 7, the single-trial oxy-Hb responses revealed a transient pattern where the mean responses remained predominantly negative during the middle stages of the session (Trials 4–6) before recovering in Trial 7. To evaluate our interpretation regarding incomplete encoding of the standard stimuli during the baseline period, we have integrated a discussion of this time-course pattern into the Discussion section.
