# Supplementary material for: An exploratory fNIRS study on directional asymmetry in segmental discrimination: Lateral–rhotic (/la–ra/) and labial–dorsal (/ba–ga/) contrasts tested in 5- and 9-month-old Japanese infants
Source: PLoS One. 2026 Sep 11;21(9):e0358044. doi: 10.1371/journal.pone.0358044 (PMC13567765; doi:10.1371/journal.pone.0358044)
Supplement: Peer review history

# Peer review: Decision Letter 0

**DOI:** 10.1371/journal.pone.0358044.r001

**Authors:** Federica Cavicchio

27 Apr 2026

Dear Dr. Akimoto,

Thank you for submitting your manuscript to PLOS ONE. After careful consideration, we feel that it has merit but does not fully meet PLOS ONE’s publication criteria as it currently stands. Therefore, we invite you to submit a revised version of the manuscript that addresses the points raised during the review process.

==============================

Dear Authors,

Thank you for submitting your manuscript to PLOS ONE. We have now received evaluations from two independent expert reviewers, and I am pleased to say that both found genuine merit in your work. Your study addresses a timely and specific question and both reviewers appreciated the clarity of your hypotheses, the appropriateness of the fNIRS methodology, and the transparency and caution with which you interpret your findings.

On the basis of these reviews, I am inviting you to submit a Major Revision. I am confident that the concerns raised are addressable and that a revised manuscript has strong potential for publication.

The reviewers' main points can be grouped around three themes:

First, both reviewers raised the question of sample size and statistical power. Given that some conditions include as few as 6–7 infants, it is difficult to determine with confidence whether the developmental difference observed between 5- and 9-month-olds reflects a genuine effect. The reviewers suggest complementing the current analyses with Bayesian approaches, which would help quantify the strength of evidence more informatively than null hypothesis testing alone under these conditions.

Second, the reviewers felt that the manuscript would benefit from a stronger theoretical grounding. In the introduction, a deeper engagement with the literature on hemispheric asymmetries and native/non-native speech perception is encouraged. In the discussion, the broader significance of the findings could be more explicitly articulated.

Third, the reviewers identified several methodological details that should be specified more clearly for reproducibility purposes, including participant inclusion criteria, stimulus characteristics, session parameters, and the completeness of the OSF repository.

Please submit your revised manuscript by Jun 11 2026 11:59PM. If you will need more time than this to complete your revisions, please reply to this message or contact the journal office at plosone@plos.org. When you're ready to submit your revision, log on to https://www.editorialmanager.com/pone/ and select the 'Submissions Needing Revision' folder to locate your manuscript file.

Please include the following items when submitting your revised manuscript:

- A letter that responds to each point raised by the academic editor and reviewer(s). You should upload this letter as a separate file labeled 'Response to Reviewers'.
- A marked-up copy of your manuscript that highlights changes made to the original version. You should upload this as a separate file labeled 'Revised Manuscript with Track Changes'
- An unmarked version of your revised paper without tracked changes. You should upload this as a separate file labeled 'Manuscript'.

If you would like to make changes to your financial disclosure, please include your updated statement in your cover letter. Guidelines for resubmitting your figure files are available below the reviewer comments at the end of this letter.

If applicable, we recommend that you deposit your laboratory protocols in protocols.io to enhance the reproducibility of your results. Protocols.io assigns your protocol its own identifier (DOI) so that it can be cited independently in the future. For instructions see: https://journals.plos.org/plosone/s/submission-guidelines#loc-laboratory-protocols. Additionally, PLOS ONE offers an option for publishing peer-reviewed Lab Protocol articles, which describe protocols hosted on protocols.io. Read more information on sharing protocols at https://plos.org/protocols?utm_medium=editorial-email&utm_source=authorletters&utm_campaign=protocols.

As the corresponding author, your ORCID iD is verified in the submission system and will appear in the published article. PLOS supports the use of ORCID, and we encourage all coauthors to register for an ORCID iD and use it as well. Please encourage your coauthors to verify their ORCID iD within the submission system before final acceptance, as unverified ORCID iDs will not appear in the published article. *Only*  the individual author can complete the verification step; PLOS staff *cannot*  verify ORCID iDs on behalf of authors.

We look forward to receiving your revised manuscript.

Kind regards,

Federica Cavicchio

Academic Editor

PLOS One

Journal Requirements:

“This work was supported by JSPS KAKENHI Grant Number 16K12451 (YA) and 16H06319, 20H05617 (RM), and by RIKEN Pioneering Research Project (Neurophysiological mechanisms toward input-driven language development). These funding sources had no role in the research. “

Please provide an amended statement that declares *all* the funding or sources of support (whether external or internal to your organization) received during this study, as detailed online in our guide for authors at http://journals.plos.org/plosone/s/submit-now.  Please also include the statement “There was no additional external funding received for this study.” in your updated Funding Statement.

Please include your amended Funding Statement within your cover letter. We will change the online submission form on your behalf.

3. Please be informed that funding information should not appear in the Acknowledgments section or other areas of your manuscript. We will only publish funding information present in the Funding Statement section of the online submission form. Please remove any funding-related text from the manuscript.

4. Your ethics statement should only appear in the Methods section of your manuscript. If your ethics statement is written in any section besides the Methods, please delete it from any other section.

5. If the reviewer comments include a recommendation to cite specific previously published works, please review and evaluate these publications to determine whether they are relevant and should be cited. There is no requirement to cite these works unless the editor has indicated otherwise.

[Note: HTML markup is below. Please do not edit.]

Reviewers' comments:

Reviewer's Responses to Questions

**Comments to the Author**

1. Is the manuscript technically sound, and do the data support the conclusions?

Reviewer #1: Yes

Reviewer #2: Partly

**********

2. Has the statistical analysis been performed appropriately and rigorously?

Reviewer #1: Yes

Reviewer #2: Yes

**********

3. Have the authors made all data underlying the findings in their manuscript fully available?

The PLOS Data policy

Reviewer #1: Yes

Reviewer #2: No

**********

4. Is the manuscript presented in an intelligible fashion and written in standard English?

Reviewer #1: Yes

Reviewer #2: Yes

**********

Reviewer #1: The study investigated infants' speech perception of a /r/ -/l/ vs /b/-/g/ contrast and explored the question of asymmetrical speech processing. Hereby, the authors tested two groups of infants, 5-month-olds and 9-month-olds, in a behavioural and fNIRS experimental setting. While behaviourally the results showed no asymmetrical perception of the /r/-/l/ contrast, the authors reported asymmetries in the 5 month-olds for the /l/- /r/ (with lower oxy-Hb in the direction from /r/ to /l/ in the right hemisphere) and /b/-/g/ (with lower oxy-Hb in the direction from /g/ to /b/ in the right hemisphere). No asymmetries were found in the group of 9-month-old infants

The study presents valuable insights into Japanese infants' perception of non-native speech sounds, contributing to our understanding of speech perception.

Introduction:

1. The introduction provides a foundation but could benefit from a more detailed discussion of the theoretical aspects for/ against asymmetrical perception.

2. Also I am missing a deeper discussion on the brain imaging studies on asymmetries and the relation to native/nonnative perception and the lateralisation in the brain

3. Please add a justification for the two ages tested in the current study

The distinction between the explorative analysis and the other behavioural and nirs experiment is not clear to me. What was the difference and why were the two groups separated? What is the difference? First, I thought it was the age, because in the behavioural experiment, only the 5-month-olds were reported, but then also the 5-month-olds were mentioned as part of the exploratory analysis.

Statistical analyses: Were the looking times normally distributed? Please consider whether log transforms might be necessary from a statistical point of view, and whether the assumption of running ANOVAs (similarly, for the NIRS analysis and the used t-tests) holds true.

Behavioural results: why would it be the case that the interstimulus interval (and the difference between the 5mo and 9mo) is relevant for the direction of the /b/-/g/ contrast but not for the /l/-/r/ contrast?

Fnirs analysis: why was only the right hemisphere analysed?

Discussion: The authors mention that the stimuli and the greater acoustic variation might be one reason why 5-month-olds cannot discriminate the /r/-/l/ contrast. Did the authors conduct some acoustic analyses for comparison?

The discussion ties together key findings, but could be strengthened by a deeper theoretical foundation. Emphasising why these findings are important beyond simply filling a gap in the literature would enhance the study’s broader relevance.

Reviewer #2: This exploratory study investigates the behavioral responses and neural basis of directional asymmetry in speech sound discrimination by measuring NIRS responses in 5- and 9-month-old infants to /la–ra/ and /ba–ga/ contrasts. The hypotheses are clearly grounded in prior literature and well motivated. Although the results are only partially consistent with previous findings, they are presented in a transparent and accessible manner. I also appreciate the authors’ appropriately cautious interpretation of the findings, particularly in light of the relatively small sample sizes and the likely limited statistical power.

Overall, I see some value in this work. The manuscript is well structured, identifies a specific gap (the neural basis of directional asymmetry) and addresses it using an appropriate method alongside a developmental comparison. Studies of this kind are particularly valuable, and empirical contributions in this area are very much welcomed, despite inherent difficulties.

Having said that, I have several concerns, especially regarding methodological specifications that could be addressed to strengthen the manuscript.

1. A major concern relates to the limited number of participants per condition, which raises questions about the robustness and interpretability of the findings. The authors themselves acknowledge this limitation in the conclusion (“Given the exploratory nature of this study, further investigations with larger sample sizes are needed…”), which is appropriate. However, this also makes it somewhat unclear what can be reliably inferred from the present data. In its current form, the study provides suggestive rather than conclusive evidence regarding the directional asymmetry. One of the more interesting aspects of the results is the apparent developmental difference, with effects observed in 5-month-olds but not in 9-month-olds. However, it is difficult to determine whether this reflects a genuine developmental change or is instead driven by limited power and/or methodological differences across age groups. The ambiguity of the behavioral results and the sample sizes (as few as 6 or 7 infants in some cases) further complicates the interpretation of the findings.

Given the practical constraints of infant research, it may not be feasible to substantially increase the sample size, which would be ideal. Alternatively, the authors should consider complementing their analyses with Bayesian approaches, which may help quantify the strength of evidence for (or against) the presence of effects, also regarding the developmental comparison. This could provide a more informative assessment than relying solely on null hypothesis significance testing under conditions of limited power.

2. For reproducibility purposes and to facilitate future comparisons, it would be valuable to clearly specify some methodological aspects:

a. Beyond standard hearing screening, did the authors adopted any inclusion criteria regarding language exposure (e.g., monolingual Japanese environment)?

b. On p. 8, when reporting on the stimuli the authors state: “After the noisy or atypical tokens were excluded, the third formant frequency transitions of /la/ and /ra/ tokens were visually checked via Praat (Phonetic Sciences, University of Amsterdam, the Netherlands), as it was the critical parameter to distinguish /l/ and /r/.” Could you please specify what was exactly seen for the transitions?

c. p. 10 “The test phase began when the average looking time in the last four habituation trials reached <65% of that in the first four habituation trials or when 28 trials were completed.” Please specify the number of participants that met one and the other criterion.

d. p. 12: “Approximately half of the infants participated in this experiment following another NIRS study.” Please clarify whether these infants were evenly distributed across the two age groups or belonged specifically to either the 5-month-old or the 9-month-old group.

e. P. 13. The authors state that “infants exhibited similar response patterns regardless of whether they had participated in the other NIRS experiment” and therefore the data were analyzed together. It would be important to clarify how this equivalence was assessed. Was this conclusion based on visual inspection of the data, or was it formally tested statistically? If statistical analyses were conducted, these should be explicitly reported.

f. P. 13 “…each infant participated in two fNIRS sessions”. Please clarify the length of each session and the time interval between these sessions. Additionally, were any analyses conducted to test for session order or carryover effects (e.g., comparing first vs. second session responses)?

g. P. 16 “ Infants with <5 available ROI channels (defined as ≥3 available trials) in any session were excluded. Please specify how many participants were excluded for this reason.

h. Plot fNIRS and Plot looking time are not available in the OSF page.

Lastly, regarding the deactivation found: in order to confirm/disconfirm their interpretation the authors could directly explore whether the responses progressively increase throughout the study in the baseline and/or decrease in oddball periods, by checking the responses in the single trials over time (without averaging them).

**********

what does this mean?). If published, this will include your full peer review and any attached files.

If you choose “no”, your identity will remain anonymous but your review may still be made public.

**Do you want your identity to be public for this peer review?**  For information about this choice, including consent withdrawal, please see our Privacy Policy

Reviewer #1: No

Reviewer #2: No

**********

[NOTE: If reviewer comments were submitted as an attachment file, they will be attached to this email and accessible via the submission site. Please log into your account, locate the manuscript record, and check for the action link "View Attachments". If this link does not appear, there are no attachment files.]

To ensure your figures meet our technical requirements, please review our figure guidelines: https://journals.plos.org/plosone/s/figures

You may also use PLOS’s free figure tool, NAAS, to help you prepare publication quality figures: https://journals.plos.org/plosone/s/figures#loc-tools-for-figure-preparation.

NAAS will assess whether your figures meet our technical requirements by comparing each figure against our figure specifications.

# Author comment: Author response to Decision Letter 1

**DOI:** 10.1371/journal.pone.0358044.r002

7 Jun 2026

Dear Reviewers,

We would like to express our sincere gratitude to the reviewers and the editor for their constructive comments and valuable suggestions on our manuscript. Their insights have been greatly helpful in significantly improving the quality and clarity of our paper.

In accordance with the reviewers’ recommendations and our re-examination of the data, we have conducted a comprehensive revision of the manuscript. The major modifications and improvements are summarized below:

Correction of Statistical Analysis: During the revision process, we re-examined our statistical approaches for both behavioral and NIRS data. For the behavioral task, considering the characteristically skewed distribution of infant looking times, we re-analyzed the data after applying a log transformation to ensure that the assumptions for parametric tests were met; note that this re-analysis did not qualitatively alter the main findings. We also identified a technical error in the t-test calculations for the NIRS ROI analysis. Consequently, while the initial ROI analysis across the three channels within the ROI no longer reached statistical significance after correcting for multiple comparisons, a post-hoc analysis focused on the middle and anterior two channels revealed a significant directional asymmetry for the /la–ra/ contrast.

Addition of Bayesian Analysis: As suggested by the reviewer, we have newly implemented a Bayesian analysis. Specifically, for the NIRS data from 5-month-old infants, a comparison of the /la/ to /ra/ and /ra/ to /la/ directions in the right middle-anterior channels provided moderate evidence for the existence of directional asymmetry. In contrast, for the 9-month-old infants, the analysis provided moderate evidence supporting the null hypothesis, indicating the absence of this asymmetry.

Stronger Theoretical Grounding: To address the core conceptual comments, we have substantially deepened the theoretical framework of the manuscript. Specifically, we have grounded our hypotheses and interpretations within the context of the Natural Referent Vowel framework and the perceptual space learning account. Furthermore, we have integrated a discussion on hemispheric asymmetries and lateralization shifts over development.

Clarification of Methodological Details: To ensure full transparency and reproducibility, we have clarified several methodological details, such as detailed information regarding the auditory stimuli. Furthermore, the related files on the Open Science Framework (OSF) have been updated and re-uploaded.

Re-English Editing: Given that these comprehensive revisions affect the entire manuscript, the entire text has been subjected to another round of professional English editing.

We believe that these extensive revisions have successfully addressed all the concerns raised by the reviewers and have significantly enhanced the theoretical and empirical value of our study. Given the comprehensive nature of these updates across the manuscript, we have not listed specific page and line numbers for each item; instead, all changes can be easily tracked in the revised manuscript with track changes, and we outline the core updates concisely in our point-by-point responses below.

Reviewer #1: The study investigated infants' speech perception of a /r/ -/l/ vs /b/-/g/ contrast and explored the question of asymmetrical speech processing. Hereby, the authors tested two groups of infants, 5-month-olds and 9-month-olds, in a behavioural and fNIRS experimental setting. While behaviourally the results showed no asymmetrical perception of the /r/-/l/ contrast, the authors reported asymmetries in the 5 month-olds for the /l/- /r/ (with lower oxy-Hb in the direction from /r/ to /l/ in the right hemisphere) and /b/-/g/ (with lower oxy-Hb in the direction from /g/ to /b/ in the right hemisphere). No asymmetries were found in the group of 9-month-old infants

The study presents valuable insights into Japanese infants' perception of non-native speech sounds, contributing to our understanding of speech perception.

Introduction:

1. The introduction provides a foundation but could benefit from a more detailed discussion of the theoretical aspects for/ against asymmetrical perception.

Response:

We appreciate this comment, which allowed us to clarify the theoretical focus of our revision. Rather than focusing on arguments for or against the mere existence of asymmetrical perception—which is generally recognized in the field—we have shifted the focus to why and how such asymmetries occur. Specifically, we have added a detailed discussion incorporating the Natural Referent Vowel framework to provide a stronger theoretical foundation for the emergence of these perceptual asymmetries.

2. Also I am missing a deeper discussion on the brain imaging studies on asymmetries and the relation to native/nonnative perception and the lateralisation in the brain

Response:

We have updated the Introduction to incorporate relevant neuroimaging findings on early speech perception and lateralization. Specifically, we have clarified that younger infants primarily recruit bilateral or right-dominant networks for acoustic-level processing of both native and non-native contrasts, and we have noted the general developmental shift toward left-hemispheric specialization as auditory cues gain linguistic significance. To keep the focus on our main topic, these neuroimaging perspectives have been integrated briefly within the context of our study.

3. Please add a justification for the two ages tested in the current study

Response:

We selected 5- and 9-month-old Japanese infants to examine the early stage of acoustic-driven, broad phonetic sensitivity at 5 months and the emerging native language specialization at 9 months. This justification has been newly added to the Introduction of the revised manuscript.

The distinction between the explorative analysis and the other behavioural and nirs experiment is not clear to me. What was the difference and why were the two groups separated? What is the difference? First, I thought it was the age, because in the behavioural experiment, only the 5-month-olds were reported, but then also the 5-month-olds were mentioned as part of the exploratory analysis.

Response:

We apologize for the confusion regarding the dataset structure. To clarify, the primary behavioral and NIRS experiments consist of the data intended for the current study (which includes both 5- and 9-month-olds for NIRS, and 5-month-olds for the behavioral task). In contrast, the post-hoc exploratory analysis was conducted by incorporating unpublished data from a preliminary experiment that involved 9-month-old Japanese infants. These data were originally collected in a preliminary study to assess the feasibility of the experimental paradigm prior to the current study. We included these additional data to increase the overall observations and explore any identifiable effects within this larger, exploratory sample. We have revised the manuscript to ensure that the distinction between the primary datasets and this exploratory sample is clearly defined.

Statistical analyses: Were the looking times normally distributed? Please consider whether log transforms might be necessary from a statistical point of view, and whether the assumption of running ANOVAs (similarly, for the NIRS analysis and the used t-tests) holds true.

Response:

We appreciate this important methodological comment. In accordance with your suggestion, and considering the characteristically skewed distribution of infant looking times, we have re-analyzed the behavioral data after applying a log transformation to ensure that the assumptions of normality for the parametric tests were met. For the NIRS analysis, the data distribution was not severely skewed like the looking times. Furthermore, we employed Welch's t-tests, which are robust against violations of statistical assumptions. Therefore, the validity of the NIRS analysis is justified without the need for additional data transformation.

Behavioural results: why would it be the case that the interstimulus interval (and the difference between the 5mo and 9mo) is relevant for the direction of the /b/-/g/ contrast but not for the /l/-/r/ contrast?

Response:

We apologize for the lack of clarity in our initial phrasing, which led to this misunderstanding. The difference in stimulus intervals between the 5-month-old and 9-month-old experiments applies to both the /b/–/g/ and /l/–/r/ contrasts, rather than specifically affecting one over the other. To prevent any further confusion, we have relocated and revised this description in the manuscript to clarify that this is a general methodological characteristic.

Fnirs analysis: why was only the right hemisphere analysed?

Response:

We regret that our previous description caused this misunderstanding. To make it explicit that we analyzed both the left and right hemispheres, we have revised the description in the manuscript as follows: "Welch’s two-tailed unpaired t-tests were conducted to compare the directional conditions for each contrast, with Bonferroni correction applied for the four comparisons (2 contrasts × 2 hemispheres)."

Discussion: The authors mention that the stimuli and the greater acoustic variation might be one reason why 5-month-olds cannot discriminate the /r/-/l/ contrast. Did the authors conduct some acoustic analyses for comparison?

Response:

We did not conduct a direct acoustic comparison with the stimuli from previous studies, as those exact tokens were unavailable. However, while previous studies typically used either synthesized stimuli or tokens from a single speaker, the present study utilized 10 tokens from 10 different speakers, making the greater acoustic variation logically apparent. To provide a clearer physical basis for our specific stimuli, we have added their acoustic profiles to the revised manuscript as follows: " Regarding the critical phonemic contrast between /la/ and /ra/, acoustic analysis confirmed that the selected /ra/ tokens exhibited a characteristic rising F3 transition (mean F3: 1944 Hz [SE = 71] at 50 ms and 2342 Hz [SE = 66] at 150 ms from onset); conversely, the /la/ tokens remained stable at a substantially higher range (mean F3: 2999 Hz [SE = 48] at 50 ms and 2958 Hz [SE = 84] at 150 ms)."

The discussion ties together key findings, but could be strengthened by a deeper theoretical foundation. Emphasising why these findings are important beyond simply filling a gap in the literature would enhance the study’s broader relevance.

Response:

We are deeply grateful for this comment, which directed us toward a much broader integration of our findings. Within the Discussion, we have significantly strengthened the theoretical foundation of our study. Specifically, we have grounded our interpretations within the context of the Natural Referent Vowel framework and the perceptual space learning account. Framing our empirical data within these theoretical perspectives has allowed us to elevate our findings beyond simply filling a literature gap, thereby clearly highlighting how our study contributes to understanding the underlying mechanisms of early speech processing.

Reviewer #2: This exploratory study investigates the behavioral responses and neural basis of directional asymmetry in speech sound discrimination by measuring NIRS responses in 5- and 9-month-old infants to /la–ra/ and /ba–ga/ contrasts. The hypotheses are clearly grounded in prior literature and well motivated. Although the results are only partially consistent with previous findings, they are presented in a transparent and accessible manner. I also appreciate the authors’ appropriately cautious interpretation of the findings, particularly in light of the relatively small sample sizes and the likely limited statistical power.

Overall, I see some value in this work. The manuscript is well structured, identifies a specific gap (the neural basis of directional asymmetry) and addresses it using an appropriate method alongside a developmental comparison. Studies of this kind are particularly valuable, and empirical contributions in this area are very much welcomed, despite inherent difficulties.

Having said that, I have several concerns, especially regarding methodological specifications that could be addressed to strengthen the manuscript.

1. A major concern relates to the limited number of participants per condition, which raises questions about the robustness and interpretability of the findings. The authors themselves acknowledge this limitation in the conclusion (“Given the exploratory nature of this study, further investigations with larger sample sizes are needed…”), which is appropriate. However, this also makes it somewhat unclear what can be reliably inferred from the present data. In its current form, the study provides suggestive rather than conclusive evidence regarding the directional asymmetry. One of the more interesting aspects of the results is the apparent developmental difference, with effects observed in 5-month-olds but not in 9-month-olds. However, it is difficult to determine whether this reflects a genuine developmental change or is instead driven by limited power and/or methodological differences across age groups. The ambiguity of the behavioral results and the sample sizes (as few as 6 or 7 infants in some cases) further complicates the interpretation of the findings.

Given the practical constraints of infant research, it may not be feasible to substantially increase the sample size, which would be ideal. Alternatively, the authors should consider complementing their analyses with Bayesian approaches, which may help quantify the strength of evidence for (or against) the presence of effects, also regarding the developmental comparison. This could provide a more informative assessment than relying solely on null hypothesis significance testing under conditions of limited power.

Response:

We are grateful for this valuable suggestion. We have conducted Bayesian analyses, which clarified the nuanced strength of our findings: for the behavioral data, the /ga/ to /ba/ change supported the null hypothesis, while the /ba/ to /ga/ change was inconclusive. For the NIRS data, the analysis provided moderate evidence for directional asymmetry in 5-month-olds (BF10 = 5.883), but moderate evidence for its absence in 9-month-olds (BF10 < 0.32). These details have been integrated into the revised manuscript.

2. For reproducibility purposes and to facilitate future comparisons, it would be valuable to clearly specify some methodological aspects:

a. Beyond standard hearing screening, did the authors adopted any inclusion criteria regarding language exposure (e.g., monolingual Japanese environment)?

Response:

Yes, all infant participants were raised in a monolingual Japanese-speaking environment. We have added this specification to the Participants section of the revised manuscript.

b. On p. 8, when reporting on the stimuli the authors state: “After the noisy or atypical tokens were excluded, the third formant frequency transitions of /la/ and /ra/ tokens were visually checked via Praat (Phonetic Sciences, University of Amsterdam, the Netherlands), as it was the critical parameter to distinguish /l/ and /r/.” Could you please specify what was exactly seen for the transitions?

Response:

To provide a clearer physical basis for our stimuli, and in response to a related comment from another reviewer, we have added these detailed acoustic profiles directly into the manuscript. Specifically, the acoustic analysis confirmed that the /ra/ tokens exhibited a characteristic rising F3 transition (Mean F3: 1944 Hz at 50 ms to 2342 Hz at 150 ms), whereas the /la/ tokens remained stable at a substantially higher range (Mean F3: 2999 Hz at 50 ms to 2958 Hz at 150 ms).

c. p. 10 “The test phase began when the average looking time in the last four habituation trials reached <65% of that in the first four habituation trials or when 28 trials were completed.” Please specify the number of participants that met one and the other criterion.

Response:

To clarify the participant numbers for each criterion, we have added this specification to the Participants s

Attachment Submitted filename: Response to reviewers.docx

# Peer review: Decision Letter 1

**DOI:** 10.1371/journal.pone.0358044.r003

**Authors:** Federica Cavicchio

26 Aug 2026

<p>An exploratory fNIRS study on directional asymmetry in segmental discrimination: lateral–rhotic (/la–ra/) and labial–dorsal (/ba–ga/) contrasts tested in 5- and 9-month-old Japanese infants

PONE-D-25-50423R1

Dear Dr. Akimoto,

We’re pleased to inform you that your manuscript has been judged scientifically suitable for publication and will be formally accepted for publication once it meets all outstanding technical requirements.

Within one week, you’ll receive an e-mail detailing the required amendments. When these have been addressed, you’ll receive a formal acceptance letter and your manuscript will be scheduled for publication.

An invoice will be generated when your article is formally accepted. Please note, if your institution has a publishing partnership with PLOS and your article meets the relevant criteria, all or part of your publication costs will be covered. Please make sure your user information is up-to-date by logging into Editorial Manager at Editorial Manager® and clicking the ‘Update My Information' link at the top of the page. For questions related to billing, please contact billing support.

If your institution or institutions have a press office, please notify them about your upcoming paper to help maximize its impact. If they’ll be preparing press materials, please inform our press team as soon as possible -- no later than 48 hours after receiving the formal acceptance. Your manuscript will remain under strict press embargo until 2 pm Eastern Time on the date of publication. For more information, please contact onepress@plos.org.

Kind regards,

Federica Cavicchio

Academic Editor

PLOS One

Additional Editor Comments (optional):

Reviewers' comments:

Reviewer's Responses to Questions

**Comments to the Author**

Reviewer #2: All comments have been addressed

**********

2. Is the manuscript technically sound, and do the data support the conclusions?

Reviewer #2: Yes

**********

3. Has the statistical analysis been performed appropriately and rigorously?

Reviewer #2: Yes

**********

4. Have the authors made all data underlying the findings in their manuscript fully available?

The PLOS Data policy

Reviewer #2: Yes

**********

5. Is the manuscript presented in an intelligible fashion and written in standard English?

Reviewer #2: Yes

**********

Reviewer #2: The manuscript has been extensively revised and, in many respects, now reads as a substantially different version. Nevertheless, the new analyses, strengthened theoretical framework, and more developed discussion have clearly improved the paper. I am satisfied that the main concerns have been adequately addressed.

Notwithstanding the exploratory nature of the study and its limitations, particularly the relatively small sample size, the authors have produced a substantially improved manuscript that makes a valuable contribution and is worthy of publication.

**********

what does this mean?). If published, this will include your full peer review and any attached files.

If you choose “no”, your identity will remain anonymous but your review may still be made public.

**Do you want your identity to be public for this peer review?**  For information about this choice, including consent withdrawal, please see our Privacy Policy

Reviewer #2: **Yes:**  Silvia Benavides-Varela

**********

# Editor report: Acceptance letter

**DOI:** 10.1371/journal.pone.0358044.r004

**Authors:** Federica Cavicchio

PONE-D-25-50423R1

PLOS One

Dear Dr. Akimoto,

I'm pleased to inform you that your manuscript has been deemed suitable for publication in PLOS One. Congratulations! Your manuscript is now being handed over to our production team.

At this stage, our production department will prepare your paper for publication. This includes ensuring the following:

* All references, tables, and figures are properly cited

* All relevant supporting information is included in the manuscript submission,

* There are no issues that prevent the paper from being properly typeset

You will receive further instructions from the production team, including instructions on how to review your proof when it is ready. Please keep in mind that we are working through a large volume of accepted articles, so please give us a few days to review your paper and let you know the next and final steps.

Lastly, if your institution or institutions have a press office, please let them know about your upcoming paper now to help maximize its impact. If they'll be preparing press materials, please inform our press team within the next 48 hours. Your manuscript will remain under strict press embargo until 2 pm Eastern Time on the date of publication. For more information, please contact onepress@plos.org.

You will receive an invoice from PLOS for your publication fee after your manuscript has reached the completed accept phase. If you receive an email requesting payment before acceptance or for any other service, this may be a phishing scheme. Learn how to identify phishing emails and protect your accounts at https://explore.plos.org/phishing.

If we can help with anything else, please email us at customercare@plos.org.

Thank you for submitting your work to PLOS One and supporting open access.

Kind regards,

PLOS One Editorial Office Staff

on behalf of

Dr. Federica Cavicchio

Academic Editor

PLOS One
